# Autophagy deficiency confers freezing tolerance in *Arabidopsis thaliana*

**DOI:** 10.1186/s12870-025-07066-9

**Published:** 2025-07-30

**Authors:** Yushi Peng, Shujuan Guo, Ben Lei, Linhui Yu, Qiuling Wang

**Affiliations:** Institute of Future Agriculture, State Key Laboratory of Crop Stress Biology for Arid Areas, Northwest A&F University, Yangling, 712100 Shaanxi People’s Republic of China

**Keywords:** Autophagy, *Arabidopsis*, Cold stress, *atg13ab*, ATG8, COR47

## Abstract

**Background:**

Plants have evolved multiple strategies to cope with the ever-changing external environment. Autophagy, as one of the crucial mechanisms involved, has been demonstrated to play a pivotal role in plant responses and adaptation to abiotic stresses. However, the precise molecular mechanisms underlying the role of autophagy in mediating cold stress remain to be fully elucidated.

**Results:**

In this study, we demonstrated that autophagy mutants presented increased freezing tolerance under both non-acclimated and cold-acclimated conditions in *Arabidopsis*. Autophagy positively regulates the expression of anthocyanin biosynthesis-related genes, thereby influencing anthocyanin accumulation in *Arabidopsis* under low-temperature conditions. Moreover, we found that cold stress directly suppresses the expression of autophagy-related genes and reduces autophagic flux in *Arabidopsis*. The RNA-seq data revealed that cold-responsive genes were pre-activated in the autophagy mutant *atg13ab* even before cold treatment. Additionally, we observed constitutive accumulation of the dehydrin protein COR47 in *atg13ab* mutant.

**Conclusions:**

Taken together, these data suggest that autophagy is a negative regulator of freezing tolerance in *Arabidopsis*.

**Supplementary Information:**

The online version contains supplementary material available at 10.1186/s12870-025-07066-9.

## Introduction

Plants face dual challenges of biotic and abiotic stresses at different growth stages. Cold stress, a critical environmental factor limiting plant geographical distribution and agricultural production, significantly inhibits plant growth and development by disrupting cell membrane integrity, inducing oxidative stress, and interfering with metabolic homeostasis [1–3]. Cold stress triggers the abnormal accumulation of denatured substances (such as misfolded proteins and damaged organelles) in plant cells [4]. If these abnormal components are not promptly cleared, they form potentially toxic aggregates, disrupt cellular metabolism, inhibit growth and development, and weaken stress resistance [5]. To address the clearance of damaged proteins under stress conditions, plants have evolved two classical degradation pathways: the autophagy-dependent degradation pathway and the ubiquitin-dependent 26S proteasome degradation pathway [6, 7]. The ubiquitin‒proteasome system (UPS) primarily targets soluble small-molecule proteins and peptides tagged with polyubiquitin chains, while autophagy is responsible for clearing insoluble protein aggregates, large protein complexes, intracellular pathogens, and damaged organelles [6, 8]. The synergistic action of these two pathways forms the core framework of the plant protein quality control network.

The autophagy process involves a membrane-independent extension structure that can encapsulate cytoplasmic components [9, 10]. This process relies on the coordinated action of various ATG (AuTophaGy-related) proteins. In plants, at least 40 ATG proteins have been identified, forming different complexes that regulate various stages of autophagy: initiation, vesicle nucleation, elongation, maturation, fusion of autophagosomes with vacuoles, and degradation of autophagosomes [11–13]. Among these, the ATG1 kinase complex (ATG1-ATG13-ATG11-ATG101) is responsible for sensing the nutritional status of cells and initiating autophagy when needed. The PI3K (phosphatidylinositol 3-kinase) complex (VPS34-VPS15-ATG6-ATG14) catalyzes the production of specific lipid signals, PI3P (phosphatidylinositol 3-phosphate), aiding in the remodeling of autophagosome membrane structures. The ATG9 complex (ATG9-ATG2-ATG18) primarily mediates lipid transport, supporting the expansion of autophagic vesicles. The two ubiquitin-like systems, ATG8-PE (phosphatidyl-ethanolamine) and ATG5-ATG12, participate in the maturation of autophagosomes, preparing them for sealing. The SNARE (soluble NSF attachment protein receptor) complex regulates the membrane fusion of autophagosomes with vacuoles, ensuring the efficient degradation of substrates. These systems work in concert to complete the entire process of autophagy, from induction to degradation [11]. Autophagy normally maintains basal levels of activity in plants [14–16], but is induced under nutrient-deficient conditions, including nitrogen, carbon, phosphorus, and sulfur [17–21]. Additionally, autophagy also serves as a critical mechanism for plants to cope with abiotic stresses, with the majority of autophagy mutants exhibiting hypersensitivity to various abiotic stresses, like salt stress [22, 23], drought stress [24], hypoxia stress [25], and heat stress [26, 27], highlighting the role of autophagy in adaptation to abiotic stresses.

Current evidence regarding the role of plant autophagy in the cold response remains contradictory. A previous study revealed that the accumulation of autophagosomes increases and that the expression of most *CaATG* genes is induced in *C. annuum* upon cold treatment [28], indicating that autophagy may positively regulate the plant cold stress response. Moreover, chilling stress significantly induces the transcription of a series of autophagy-related genes, such as *ATG2*, *ATG6*, and *ATG8*, and enhances the autophagic flux in tomato. Increased autophagic activity thus improves the cold tolerance of plants [29]. Further research revealed that among this series of cold-induced tomato *ATG* genes, *ATG18a* is also included. The mutant of *ATG18a* exhibited sensitivity to cold treatment, along with decreased autophagosome formation and increased accumulation of ubiquitinated proteins [30]. Cold also induces the expression of selective autophagy receptors such as *NBR1a* (a neighbor of BRCA1), *NBR1b*, and *SEC62* in tomato, and silencing these selective autophagy receptors affects the generation of autophagy, leading to compromised cold tolerance [31]. Additionally, *ATG13* is upregulated in response to cold stress in *M. sativa*, and compared with wild-type plants, transgenic tobacco overexpressing *MsATG13* enhanced cold tolerance compared to wild-type plants due to the upregulation of other *ATGs* that are necessary for autophagosome production under cold stress conditions [32]. In *S. melongena*, the transcription factor SmWRKY26 enhances cold tolerance by promoting the expression of multiple autophagy-related genes and stimulating autophagosome formation under cold stress conditions [33]. These all suggest the positive role of autophagy in plant resistance to cold stress. However, some studies indicate that autophagy in plants is restricted under low-temperature conditions. Compared to untreated controls, cold-treated *Arabidopsis* plants showed reduced transcription of autophagy-related genes. Concurrently, levels of the autophagic marker ATG8 decreased, while accumulation of the autophagy substrate NBR1 protein increased [34, 35]. These observations suggest that autophagy is inhibited or impaired under cold stress. A recent study revealed that autophagy is dispensable for cold acclimation and freezing tolerance in *Arabidopsis*, *atg5-1* and *atg10-1* mutants exhibited normal freezing tolerance, cold-regulated gene expression showed no significant differences between the *atg* mutants and wild- type as well as autophagy was rarely induced by cold exposure [36]. These conflicting results suggest the complex role of autophagy in plant cold stress responses, as well as the functional divergence of autophagy across species under stress conditions.

In this study, we found that autophagy mutants of *Arabidopsis* exhibited enhanced tolerance to freezing. Cold treatment suppressed autophagy activity, as evidenced by reduced transcription of autophagy-related genes and decreased ATG8 protein abundance. Furthermore, cold-responsive genes showed constitutive expression in autophagy mutants, and the cold-regulated protein COR47 exhibited greater stability in these mutants. These findings indicate that autophagy acts as a negative regulator for plant cold stress adaptation in *Arabidopsis*.

## Results

### Autophagy negatively regulates freezing tolerance in* Arabidopsis*

**Fig. 1:**
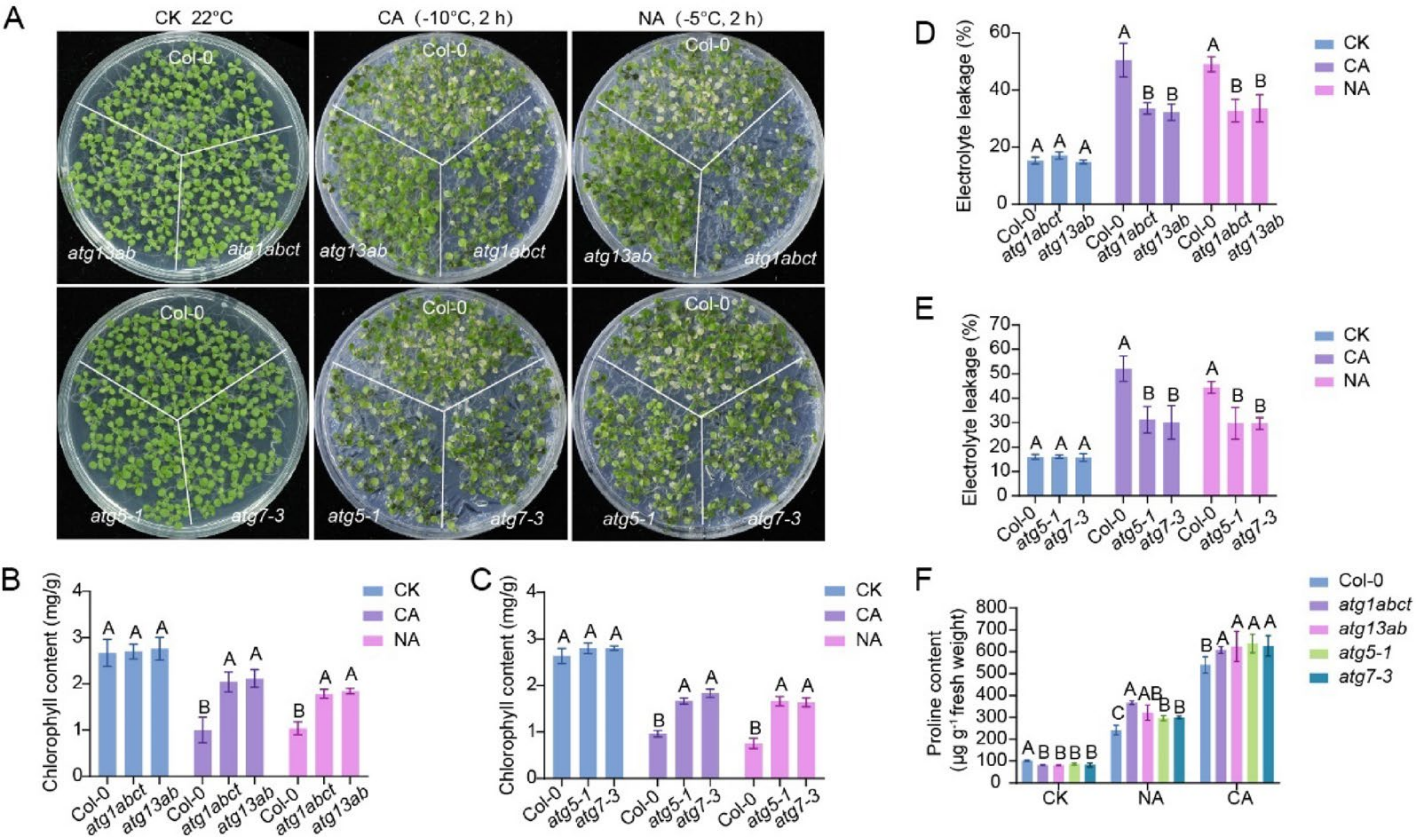
Autophagy mutants exhibit tolerance to freezing stress in *Arabidopsis*. **A** Freezing phenotype of the autophagy mutants under cold-acclimated (CA) and non-acclimated (NA) conditions. 14-day-old seedlings grown on half-strength MS (1/2 MS) plates at 22 °C were treated at − 10 °C for 2 h after pre-treatment at 4 °C for 2 days (CA) or were directly treated at − 5 °C for 2 h (NA).** B**, **C** Chlorophyll contents of the seedlings shown in (**A**).** D**, **E** Ion leakage of the seedlings shown in (**A**). **F** Proline content in WT and autophagy mutants at 22 °C, as well as cold treatment under CA or NA conditions. In (**B**–**F**), each bar represents the mean ± SD of three independent experiments. Different letters above the columns indicate significant differences determined using one-way ANOVA with Tukey’s multiple comparison analyses (*P* < 0.05)

To explore the contributions of autophagy to freezing tolerance in *Arabidopsis*, various autophagy mutants (*atg1abct*, *atg13ab*, *atg5-1*, and *atg7-3*) were collected as described previously [19, 37]. Fourteen-day-old seedlings were subjected to freezing treatment. Under cold-acclimated (CA) conditions, these mutants displayed enhanced freezing tolerance compared to the wild-type plants (reflected by higher chlorophyll content) (Fig. 1A–C). Next, we examined ion leakage, which is an indicator of stress-induced plasma membrane damage in the autophagy mutants. Ion leakage in the mutants under CA conditions was consistently lower than that in the wild type (Fig. 1D, E). Since autophagy is implicated in plant stress memory, autophagy mutants that undergo heat priming retain thermotolerance memory and exhibit increased resistance to subsequent heat stress [27]. We therefore investigated whether non-acclimated (NA) autophagy mutants maintained improved freezing tolerance. Consistent with their performance under CA conditions, these autophagy mutants still displayed higher chlorophyll contents and lower ion leakage rates without cold acclimation (Fig. 1A–E). Furthermore, proline, a well-documented stress-protective metabolite in plants [38], was found to accumulate at higher levels in autophagy mutants than in wild-type plants following cold treatment, observed under both NA and CA conditions (Fig. 1F). These findings collectively demonstrate that autophagy serves as a negative regulator of freezing tolerance in *Arabidopsis*.

### Autophagy regulates the accumulation of anthocyanins in *Arabidopsis* under chilling conditions

**Fig. 2:**
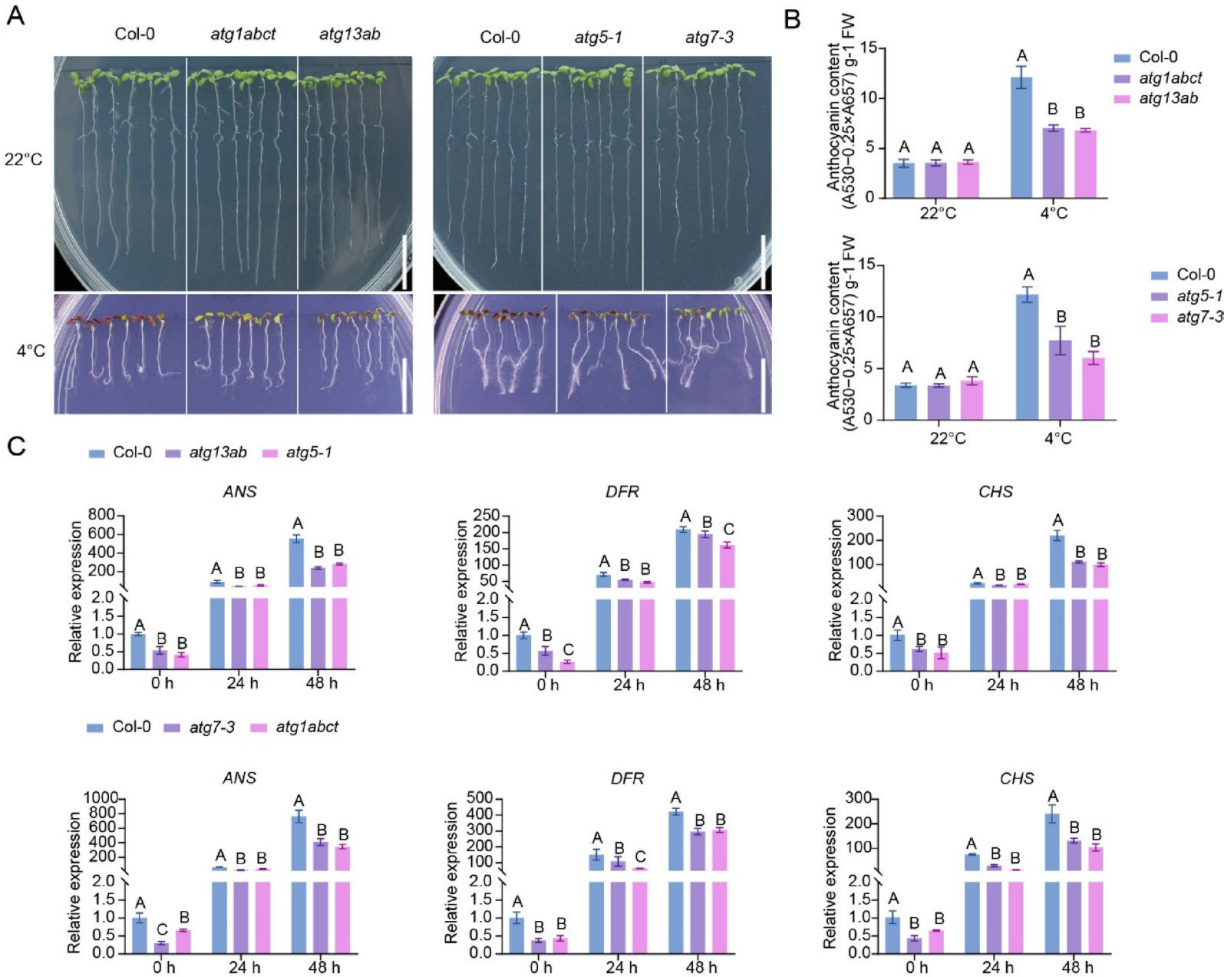
Autophagy regulates anthocyanin accumulation in *Arabidopsis* under chilling conditions. **A** WT and autophagy mutants were grown at 22 °C or 4 °C. Five-day-old seedlings were transplanted to 1/2 MS medium containing 1% agar and grown at 22 °C for an additional 8 d or 4 °C for an additional 45 d before photographs were taken. Bar = 1 cm. **B** Anthocyanin content of the seedlings shown in (**A**), FW, fresh weight. **C** qRT-PCR analysis of the expression of selected anthocyanin biosynthetic genes in 14-day-old seedlings of WT, *atg1abct*, *atg13ab*, *atg5-1*, and *atg7-3* mutants upon 4 °C treatment for indicated times. *Ubiquitin10* (*UBQ10*) was used as an internal control. In (**B**, **C**), each bar represents the mean ± SD of three independent experiments. Different letters above the columns indicate significant differences determined using one-way ANOVA with Tukey’s multiple comparison analyses (*P* < 0.05)

To validate the function of autophagy under chilling conditions in plants, the wild type and all the autophagy mutants were grown at 22 °C and 4 °C under normal photoperiod conditions (16 h/light and 8 h/dark), respectively. No significant differences were detected among the plants grown at 22 °C (Fig. 2A). However, under continuous growth at 4 °C for 45 days, the wild-type plants exhibited a noticeable accumulation of anthocyanins in their cotyledons as previously described [39], while the cotyledons of the mutants still maintained a distinct green color (Fig. 2A, B). As *DFR* (dihydroflavonol 4-reductase), *CHS* (chalcone synthase), and *ANS* (anthocyanin synthase) are key enzymes in anthocyanin biosynthesis [40, 41], further analysis of the expression of these genes in different plants revealed that the expression of *DFR*, *CHS*, and *ANS* was suppressed in the autophagy mutants *atg1abct*, *atg13ab*, *atg5-1*, and *atg7-3* both before and after cold treatment (Fig. 2C). These results indicate that autophagy negatively regulates anthocyanin accumulation in *Arabidopsis* under chilling conditions and that anthocyanins likely do not directly contribute to the increased cold tolerance observed in autophagy mutants.

### Cold inhibits the expression of autophagy-related genes in *Arabidopsis*

**Fig. 3:**
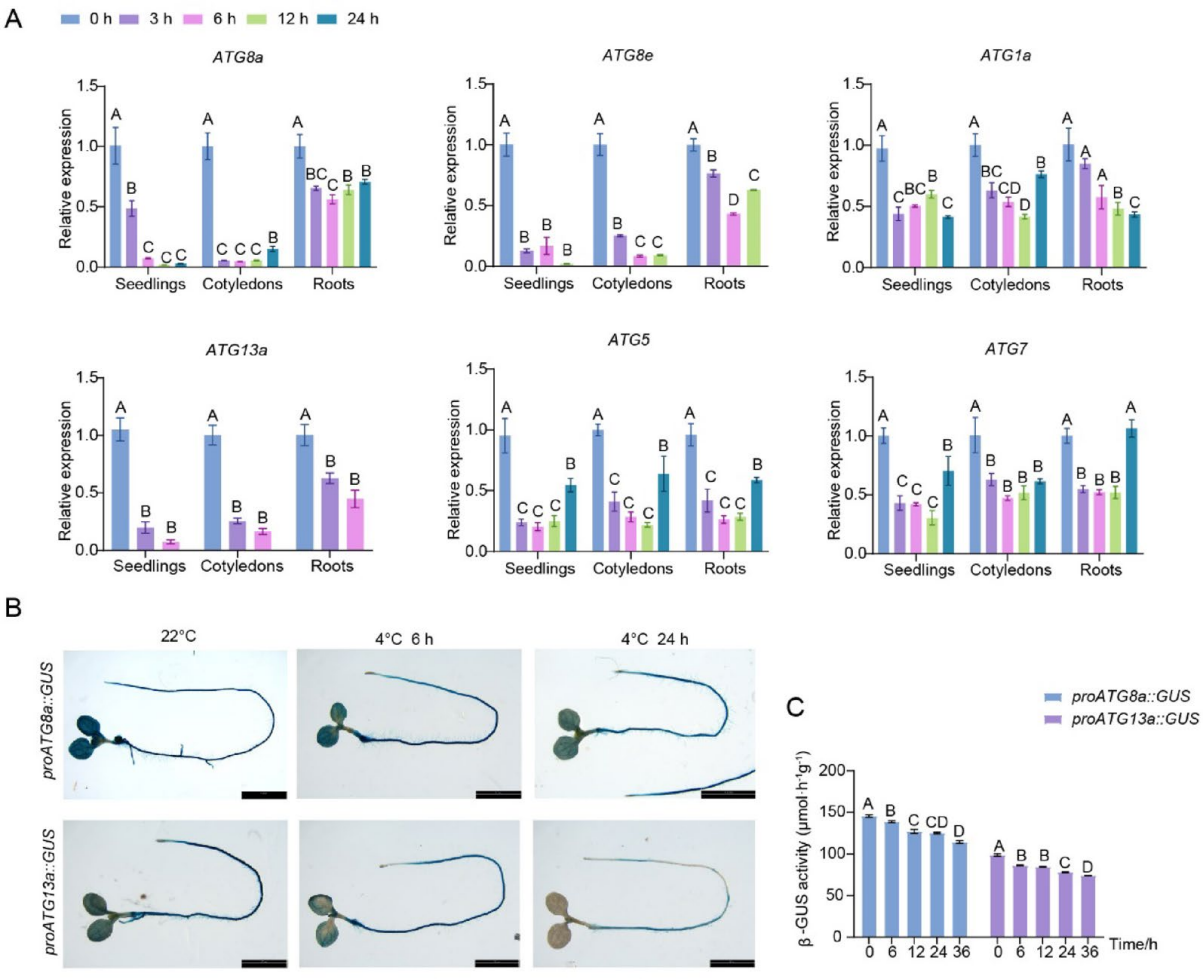
Cold inhibits the expression of autophagy-related genes in *Arabidopsis*. **A** qRT-PCR analysis of the expression of selected autophagy-related genes in Col-0 (seedlings, cotyledons, and roots) upon 4 °C treatment for the indicated times. *Ubiquitin10* (*UBQ10*) was used as an internal control. **B** GUS histochemical staining of the 10-day-old transgenic seedlings expressing *GUS* gene driven by *ATG8a* and *ATG13a* promoters upon 4 °C treatment for the indicated times. Bars = 7 mm. **C** Analysis of GUS enzyme activity in seedlings of B after cold treatment for the indicated times. In (**A**, **C**), each bar represents the mean ± SD of three independent experiments. Different letters above the columns indicate significant differences determined using one-way ANOVA with Tukey’s multiple comparison analyses (*P* < 0.05)

To investigate how cold affects autophagy in *Arabidopsis*, we examined the transcription of autophagy-related genes after treatment at 4 °C under normal photoperiod conditions. Cold treatment reduced the expression of a series of autophagy-related genes in seedlings, including *ATG8a*, *ATG8e*, *ATG1a*, *ATG13a*, *ATG5*, and *ATG7* (Fig. 3A). Since leaves are the primary organs for sensing and responding to cold stress, we analyzed the responses of these *ATG* genes in cotyledons and roots separately. Cold reduced the expression of these genes in both tissues, but the response in roots was slightly delayed compared to cotyledons, particularly *ATG8a*, *ATG8e*, and *ATG13a* (Fig. 3A). To further characterize the transcriptional regulation of autophagy-related genes, we generated stable transgenic lines by fusing the promoter regions upstream of the *ATG8a* and *ATG13a* transcription start sites to the β-glucuronidase (GUS) reporter gene. Histochemical GUS staining revealed that under normal growth conditions, both the *ATG8a* and *ATG13a* promoters drove strong reporter gene expression in the cotyledons and roots of *Arabidopsis* seedlings. However, following cold treatment at 4 °C, we observed a significant reduction in GUS expression for both genes after 6 h of cold exposure, and this suppression was particularly pronounced in cotyledons, matching the qRT-PCR results. The suppression became more apparent after 24 h of cold treatment (Fig. 3B). GUS enzyme activity analysis further supported these findings (Fig. 3C). These results suggest that low temperatures inhibit the transcription of autophagy genes in *Arabidopsis*.

### Autophagic flux is reduced upon cold treatment in *Arabidopsis*

**Fig. 4:**
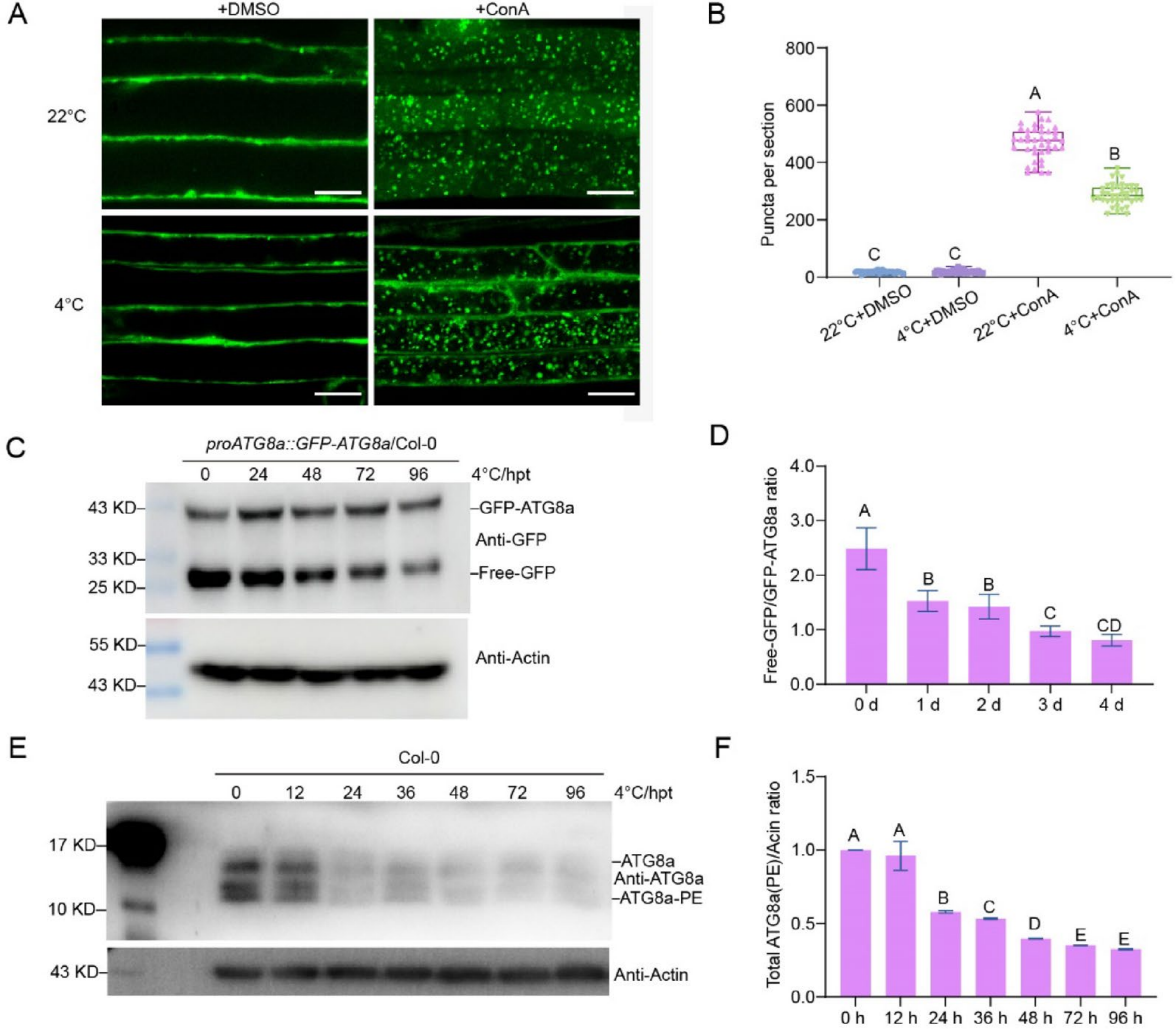
Autophagic flux is reduced upon cold treatment. **A** Deposition of GFP-ATG8a-labeled autophagic bodies inside the central vacuole of WT at 22 °C or 4 °C. Seedlings of *proATG8a::GFP-ATG8a*/Col-0 grown on 1/2 MS agar medium for 5 days at 22 °C were transferred to 1/2 MS liquid medium and either continued culturing at 22 °C for 12 h (control), or exposed to 4 °C for 12 h under a standard photoperiod. Seedlings were then allowed to recover for 6 h at 22 °C. Subsequently, either DMSO (control) or 0.5 μM ConA was added to the liquid medium, followed by further incubation at 22 °C for 4 h before microscopy observation. Concanamycin A (ConA), 0.5 μM. Scale bars, 10 µm.** B** Quantification of the number of autophagic bodies shown in (A). Four different images in the root elongation zone were photographed per seedling and the number of puncta in each image was counted and averaged. A total of ten seedlings were observed per treatment. Different letters represent significant differences at *P* < 0.05 (one-way ANOVA and Tukey’s multiple comparison test, *n* = 40). **C** Immunoblotting analysis showing the processing of GFP-ATG8a in WT upon 4 °C treatment for the indicated times. **D** Relative ratio of free GFP to GFP-ATG8a shown in (**C**), as quantified by ImageJ. **E** Immunoblot detecting the ATG8 lipidation level in Col-0 upon 4 °C treatment for the indicated times. **F** Relative intensity of total ATG8 protein normalized to the loading control Actin shown in (E). In (**D**, **F**), each bar represents the mean ± SD of three independent experiments. Different letters above the columns indicate significant differences determined using one-way ANOVA with Tukey’s multiple comparison analyses (*P* < 0.05). All protein experiments were repeated at least 3 times with similar results. Actin was used as a protein loading control. hpt, hours post-treatment

The observed decrease in autophagy-related gene expression following cold treatment led us to examine whether autophagic activity might also be affected. To investigate this possibility, we employed the *pATG8a::GFP-ATG8a*/Col-0 transgenic line, which has been established as a reliable marker for autophagosome visualization [42]. In the presence of concanamycin A (ConA), an autophagy inhibitor that stabilizes autophagic bodies [17, 18], it was observed that a noticeable reduction in GFP-ATG8a-labeled autophagosomes occurred after cold treatment compared to control conditions (Fig. 4A, B). To further assess autophagic activity, we monitored the release of free GFP from the GFP-ATG8a fusion protein, a well-documented method for tracking autophagic transport [18]. Consistent with the microscopy results, free GFP levels were reduced upon cold treatment (Fig. 4C, D). The lipidation of ATG8 and the cleavage efficiency of the GFP-ATG8 fusion protein are commonly used indicators of autophagic flux [18, 43]. After cold exposure, both lipidated and non-lipidated forms of ATG8 showed reduced accumulation after cold exposure (Fig. 4E, F). These results demonstrate that cold treatment leads to inhibition of autophagic flux in *Arabidopsis*.

### Differentially expressed gene (DEG) analysis between Col-0 and the *atg13ab* mutant

**Fig. 5:**
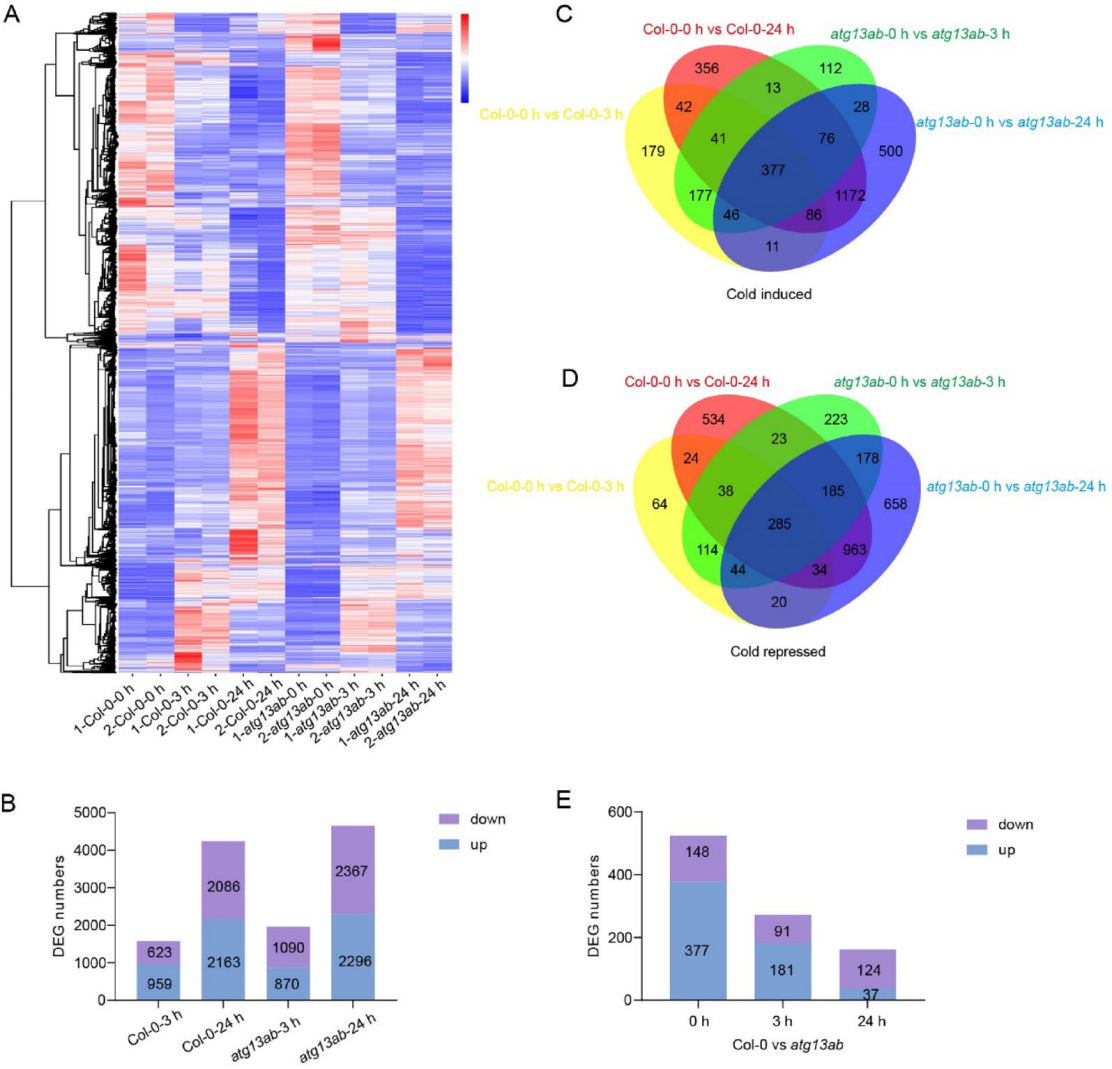
Differentially expressed gene (DEG) analysis between Col-0 and *atg13ab* mutant. **A** Heatmap of DEGs in Col-0 and *atg13ab* before or after cold treatment. The genes analyzed included the overlap of DEGs in *atg13ab* as compared with Col-0. DEGs were defined as FDR < 0.05 &|log2 (foldchange)|≥ 1. **B** Number of up- and down-regulated DEGs between cold-treated for 3 or 24 h venus untreated (0 h of cold treatment) samples in Col-0 and *atg13ab*.** C**, **D** The cold induced (**C**) or repressed (**D**) gene expression in Col-0 and *atg13ab*. Fourteen-day-old seedlings were treated at 4 °C for 0, 3, and 24 h for RNA-Seq assay. Each sample was compared with 0 h time point to choose the cold induced (log2 ≥ 1, FDR ≤ 0.05) or repressed (log2 ≤ 1, FDR ≤ 0.05) genes. The differentially expressed genes were picked out to draw the Venn diagram. **E** Number of up- and down-regulated DEGs between Col-0 and *atg13ab* at the indicated hours of cold treatment

To further investigate the effect of autophagy on the transcriptome profile, we performed RNA-seq analysis of 14-day-old wild-type and *atg13ab* seedlings treated at 4 °C for 0, 3, or 24 h. Two independent experiments were carried out (Fig. 5A), and the differentially expressed genes were analyzed via HTSeq and DESeq2 software. At 3 h after cold treatment, 959 genes were up-regulated (log2 ≥ 1, FDR ≤ 0.01), and 623 genes were down-regulated (log2 ≤ 1, FDR ≤ 0.01) in the wild-type. When cold treatment was extended to 24 h, 2163 genes were up-regulated and 2086 genes were down-regulated (Fig. 5B). In the *atg13ab* mutant, the up-regulated genes numbered 870 and 2296, respectively, at 3 h or 24 h of cold treatment, and the down-regulated genes numbered 1090 and 2367, respectively (Fig. 5B). Moreover, a total of 1999 cold-induced genes and 1706 cold-repressed genes were identified in both the wild-type and *atg13ab* mutant (Fig. 5C, D). These results suggest that a large number of cold regulate genes are not affected in the *atg13ab* mutant under cold stress.

For both Col-0 and *atg13ab*, the number of DEGs induced at 24 h was significantly greater than that induced at 3 h (Fig. 5B), indicating that cold treatment for 24 h caused substantial transcriptional rearrangement within the plant. Additionally, it was observed that a large number of DEGs were found in both wild-type and *atg13ab* even under normal growth conditions, while the number of DEGs in Col-0 and *atg13ab* decreased as the duration of cold treatment extended (Fig. 5E). Compared with Col-0, untreated *atg13ab* had 525 DEGs, with 377 genes up-regulated and 148 genes down-regulated; at 3 h of cold treatment, there were a total of 272 DEGs in Col-0 and *atg13ab*, with 181 upregulated and 91 downregulated; and at 24 h of cold treatment, these DEGs numbered only 161, including 37 up-regulated and 124 down-regulated (Fig. 5E). These results indicated that cold stress reduced the difference in gene expression profiles between Col-0 and the *atg13ab* mutant, suggesting that autophagy deficiency also has a substantial effect on gene expression but that this effect occurs primarily in untreated plants.

### GO analysis of DEGs between unstressed Col-0 and the *atg13ab* mutant

**Fig. 6:**
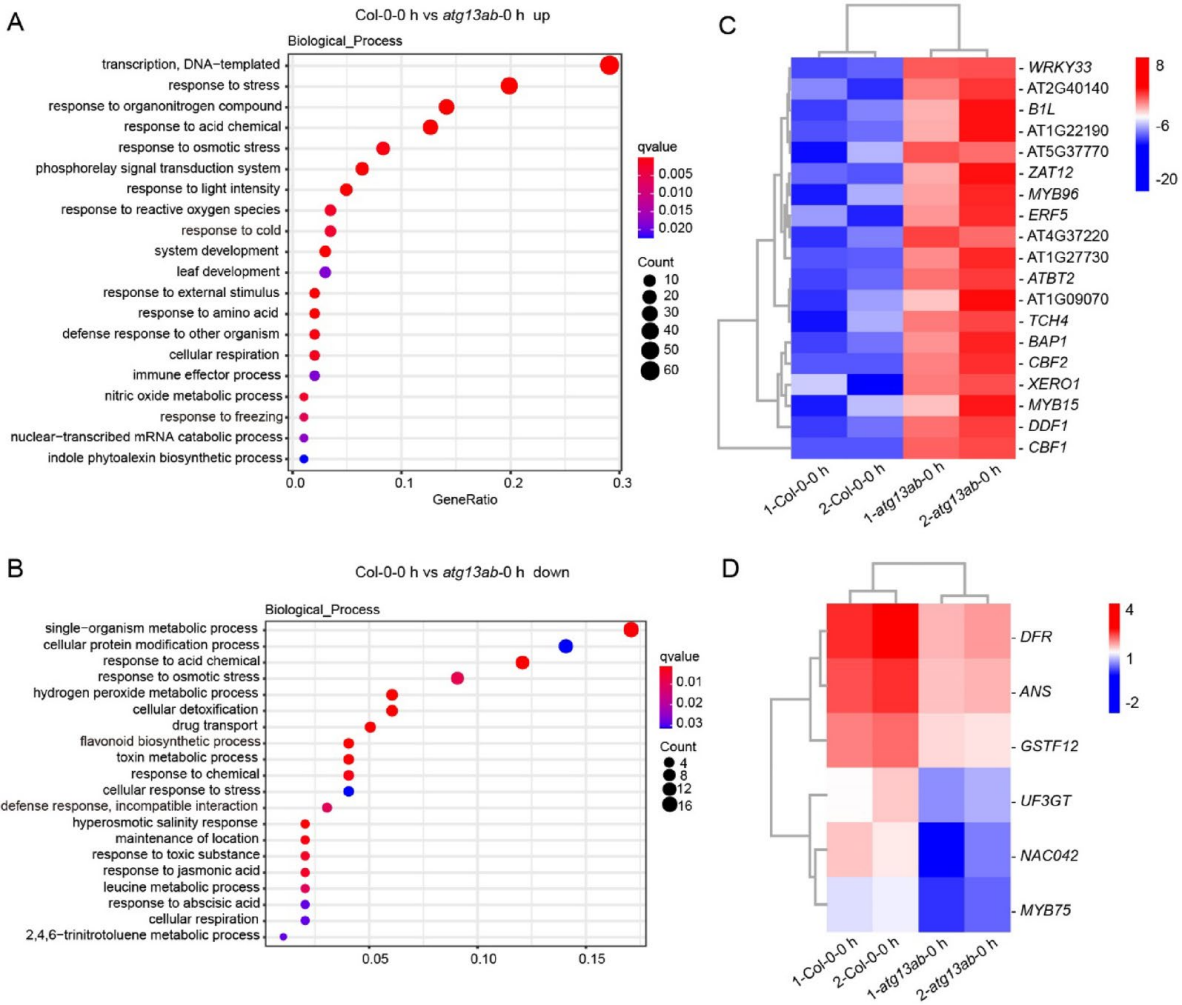
GO enrichment analysis of DEGs between Col-0 and *atg13ab* mutant at 0 h of cold treatment. **A, B** The enrichment analysis of up-regulated (**A**) and down-regulated (**B**) DEGs was performed using the GO term that describes biological process. Fold change ≥ 2, *P*‐value (FDR) < 0.01. **C** Clustering display of the induced cold and freezing‐related DEGs in *atg13ab* compared to Col-0 before cold treatment. **D** Clustering display of the repressed flavonoid biosynthetic‐related DEGs in *atg13ab* compared to Col-0 before cold treatment

The wild-type and *atg13ab* mutant displayed a large difference in gene expression profiles before cold treatment (Fig. 5E). To understand the effects of autophagy on plant gene expression under normal temperature, we first performed GO enrichment analysis to assign biological processes to the identified DEGs between Col-0 and *atg13ab* at 0 h of cold treatment (Fig. 6A, B). Among the up-regulated DEGs in the *atg13ab* mutant plants, those involved in transcription and DNA replication, the stress response, the organonitrogen compound and acid chemical response, and the osmotic stress response were enriched (Fig. 6A). These findings suggest that autophagy deficiency may lead to dysregulation of the cell cycle and that intracellular nitrogen metabolism, the cytoplasmic pH, and osmotic pressure may be altered. Surprisingly, we found that genes responsive to cold and freezing were also enriched (Fig. 6A), including early cold-responsive genes such as the CBF (C-repeat binding factor)/DERB1 (dehydration-responsive element binding) transcription factors *CBF1*, *CBF2*, and *DDF1* (dwarf and delayed flowering 1) [44, 45], along with well-characterized cold-inducible genes such as *B1L1* (BYPASS1-LIKE), *ZAT12* (ZINC FINGER OF ARABIDOPSIS THALIANA12), and *MYB15* [46–48] (Fig. 6C), indicating that the cold signaling pathway is activated in the *atg13ab* mutant under basal conditions. This may be the primary reason for the tolerance of the autophagy mutant to cold stress.

Further analysis of the down-regulated DEGs in Col-0 and *atg13ab* under unstressed conditions revealed that these genes were enriched primarily in pathways related to single-organism metabolism, cellular protein modification, hydrogen peroxide metabolism, cellular detoxification, and drug transport (Fig. 6B). This suggests that the mutant appears to exhibit changes in basal metabolism, protein homeostasis, oxidative stress response, detoxification capacity, and xenobiotic resistance. We also observed that genes involved in flavonoid biosynthesis were downregulated in the mutant, including the aforementioned anthocyanin biosynthesis-related genes *ANS* and *DFR*, as well as the key anthocyanin regulatory transcription factors *MYB75* and *NAC042* [49, 50] (Fig. 6D), which are consistent with our qRT-PCR results from previous chilling experiments (Fig. 2C). Additionally, the plant defense response and incompatible interaction-related genes were down-regulated in the mutant (Fig. 6B), suggesting that the mutant may have defects in plant immunity.

### GO analysis of DEGs between Col-0 and the *atg13ab* mutant in response to cold stress

As described previously, cold triggers extensive transcriptional reprogramming in *Arabidopsis* (Fig. 5). To systematically investigate how cold affects plant gene expression, we performed GO enrichment analysis of DEGs in Col-0 plants under cold treatment. Our analysis revealed that after 3 h and 24 h of cold exposure, the up-regulated DEGs were enriched predominantly in pathways related to transcription, DNA replication, organonitrogen compound, acid chemical, and metal ion responses; cold and osmotic stress response; and RNA processing, or metabolism (Supplementary Fig. 1A). Conversely, the down-regulated DEGs were enriched primarily in pathways such as transcription, DNA replication, single-organism metabolism, protein modification, hormone responses, and lipid/fatty acid metabolism (Supplementary Fig. 1B). Notably, we observed up-regulation of salicylic acid-related and fungal defense-related genes in Col-0 (Supplementary Fig. 1A), indicating that cold may activate immunity-related pathways.

**Fig. 7:**
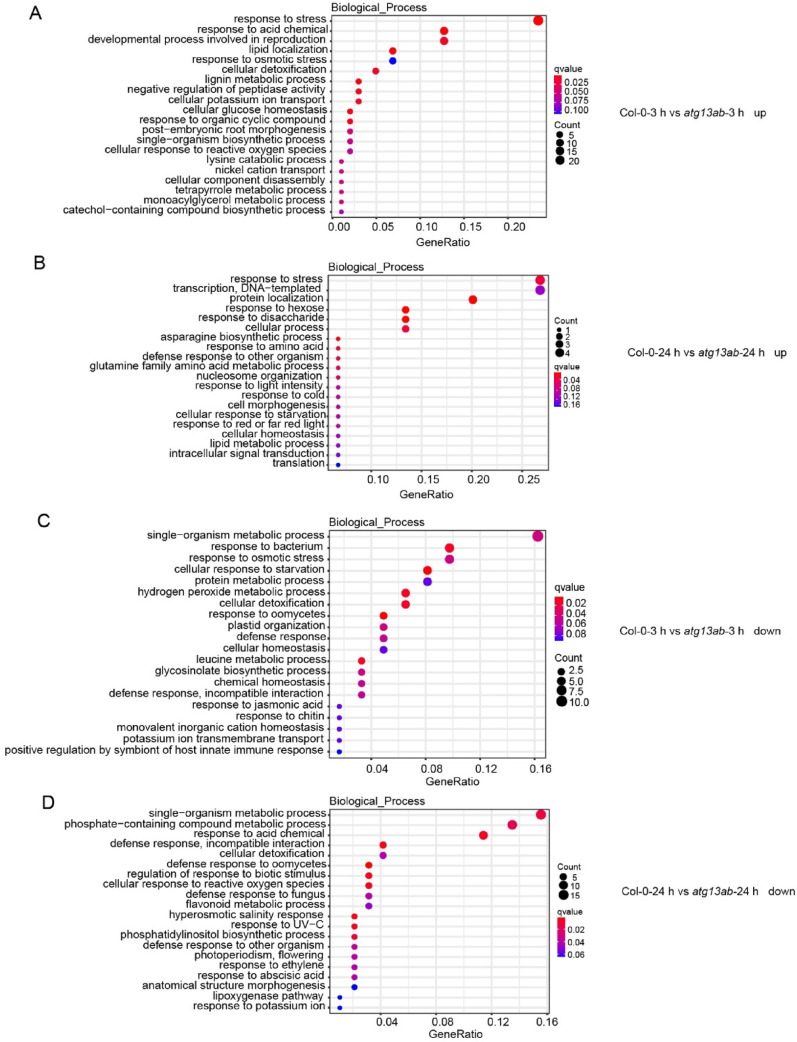
GO enrichment analysis of DEGs between Col-0 and *atg13ab* mutant upon cold treatment at 3 h and 24 h. The enrichment analysis of up-regulated at 3 h (**A**), down-regulated at 3 h (**B**), up-regulated at 24 h (**C**), and down-regulated at 24 h (**D**)

We further analyzed the DEGs between Col-0 and *atg13ab* after cold treatment. The up-regulated genes showed enrichment in processes including stress response, transcription and DNA replication, responses to acids/chemicals/hexoses/disaccharides, and lipid/protein trafficking (Fig. 7A, B). These findings collectively indicate that autophagy deficiency impacts multiple cellular processes under cold stress, potentially through dysregulation of stress signaling and genomic maintenance mechanisms, metabolic reprogramming to maintain energy homeostasis, and membrane remodeling via altered lipid distribution. Notably, a subset of cold-responsive genes maintained elevated expression in *atg13ab* even after 24 h of cold exposure (Fig. 7B), suggesting their potential role in conferring cold stress tolerance. Intriguingly, while baseline expression of immune-related genes was already reduced in untreated *atg13ab* mutants (Fig. 6B), cold treatment for 3 h and 24 h led to pronounced down-regulation of defense response pathways against bacterial and fungal pathogens (Fig. 7C, D). This progressive suppression of immune-related gene expression implies compromised pathogen defense capacity in *atg13ab* during cold stress conditions.

**Fig. 8:**
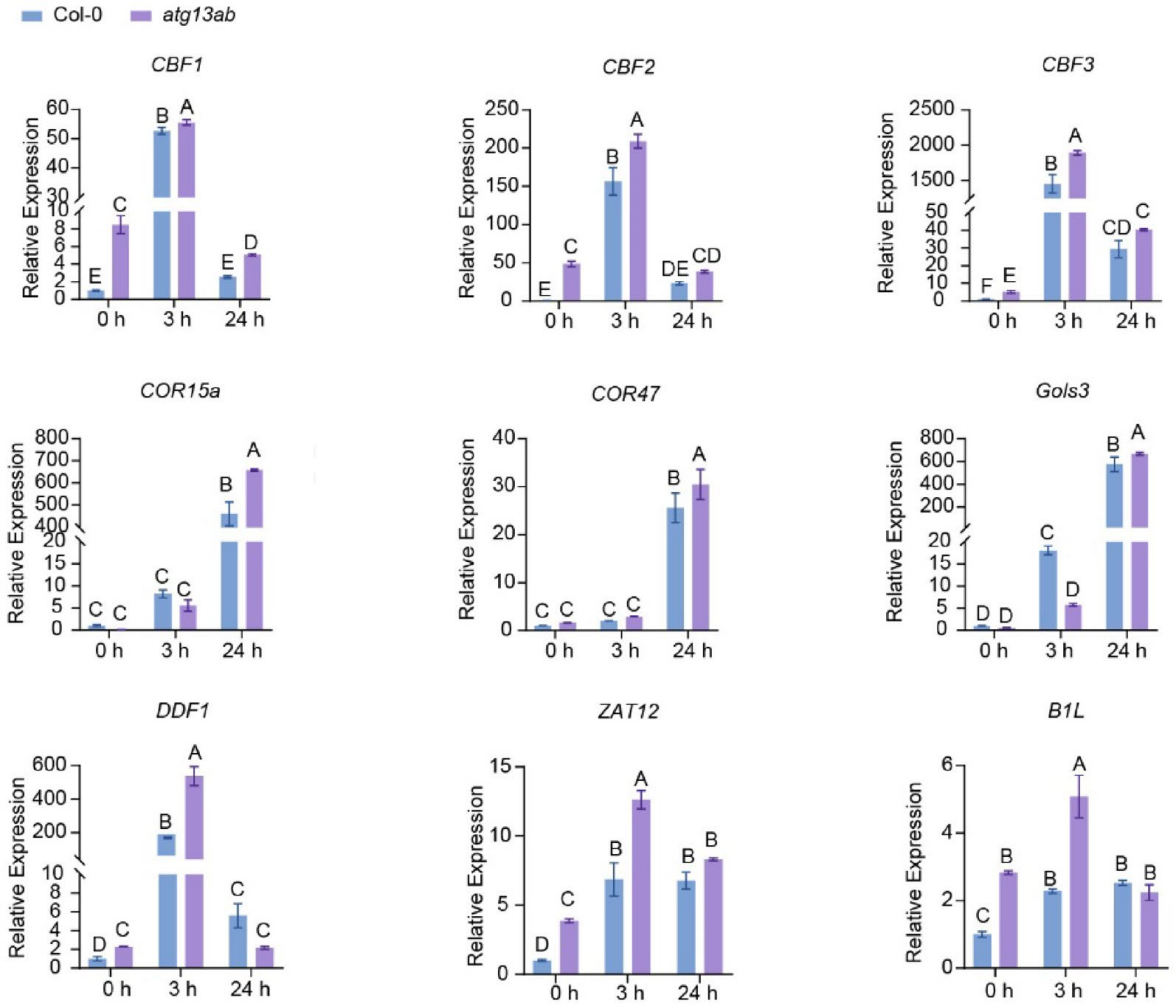
qRT-PCR analysis of the expression levels of cold-related genes in *atg13ab* compared to Col-0 before and after cold treatment. *UBQ10* was used as a reference gene. Each bar represents the mean ± SD of three independent experiments. Different letters represent significant differences at* P* < 0.05 (one-way ANOVA and Tukey’s multiple comparison test)

To validate the RNA-seq data, cold-associated genes, including *CBF1/2/3*, *COR15a* (COLD-REGULATED 15a), *COR47*, *Gols3* (galactinol synthase 3), *DDF1*, *B1L*, and *ZAT12,* were selected for qRT-PCR [51]. Consistent with the RNA-seq data, the expression of the *CBFs*, *DDF1*, *B1L*, and *ZAT12* was elevated in the mutant under unstressed conditions (Fig. 8). After 3 h of cold stress, *CBFs*, *DDF1*, *B1L*, and *ZAT12* in *atg13ab* still maintained high expression levels (Fig. 8)‌. Although the basal expression of late cold-responsive genes (*COR15a*, *COR47*, *Glos3*) was comparable to that of the wild type under normal conditions, their induction in the mutants was higher than in the wild type after 24 h of cold treatment (Fig. 8). These results suggest that autophagy deficiency leads to activation of the cold signaling pathway in *Arabidopsis*.

### COR47 accumulated in the autophagy mutant *atg13ab*

**Fig. 9:**
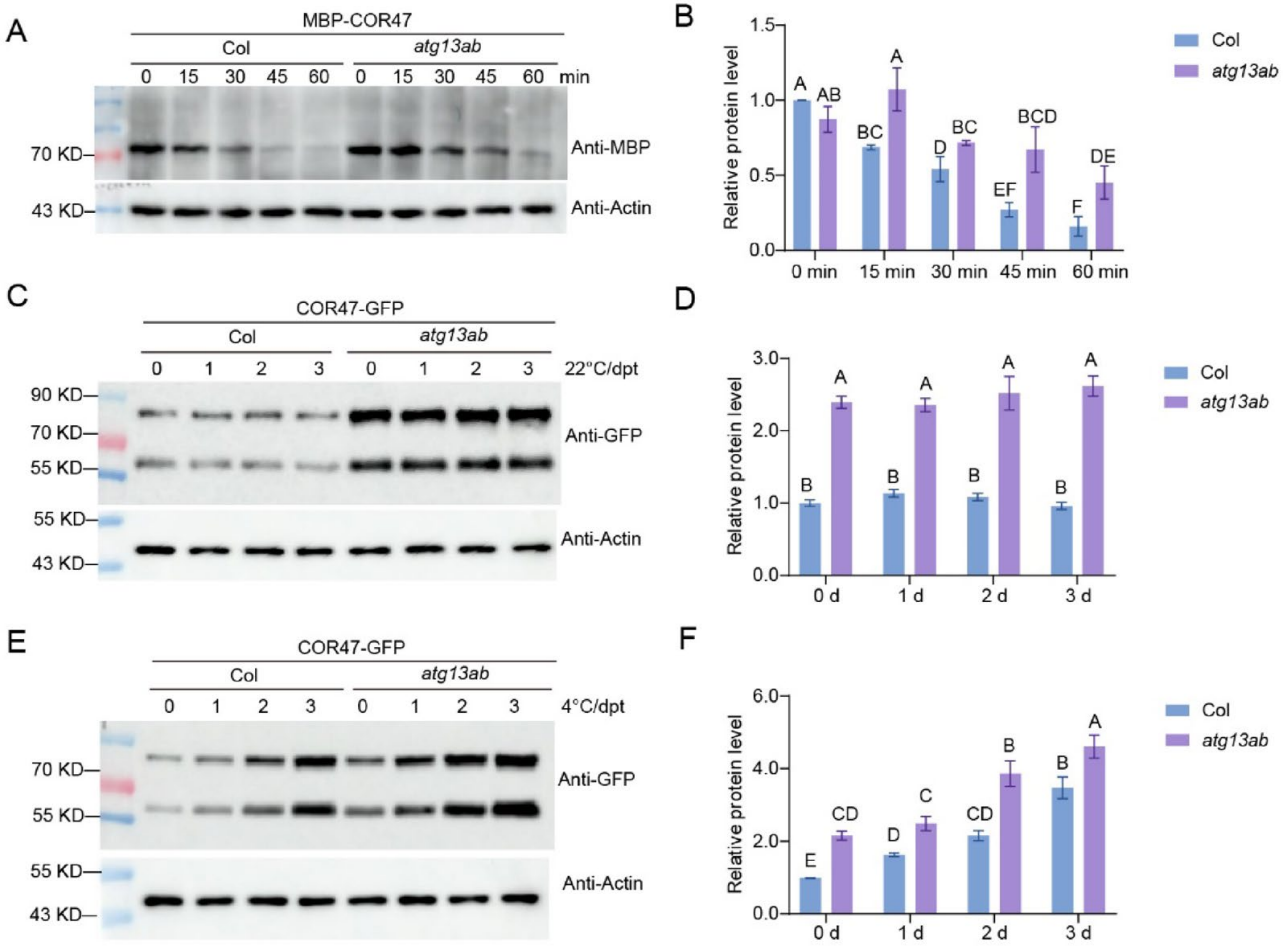
COR47 accumulated in the autophagy mutant *atg13ab*. **A** and **B** Degradation of COR47 was inhibited in the autophagy mutant *atg13ab* in the cell-free degradation assay. Recombinant purified MBP-COR47 was incubated in equal amounts of total proteins extracted from 10-day-old Col-0 and *atg13ab* seedlings in the presence of 1 mM ATP with 5 μM MG132. MBP-COR47 was detected with anti-MBP antibody. Relative intensity of MBP-COR47 normalized to the loading control Actin was shown in (B). **C** and **D** The stability of COR47-GFP in Col-0 and *atg13ab* at 22 °C. For C, 10-day-old Col-0 and *atg13ab* expressing *proCOR47::COR47-GFP* were maintained at 22 °C. Total proteins were extracted and subjected to immunoblotting using an anti-GFP antibody. **E** and **F** The stability of COR47-GFP in Col-0 and *atg13ab* at 4 °C for the indicated time. In (**B**, **D**, **F**), each bar represents the mean ± SD of three independent experiments. Different letters represent significant differences at *P* < 0.05 (one-way ANOVA and Tukey’s multiple comparison test). All protein experiments were repeated at least 3 times with similar results. hpt, hours post-treatment

Given the up-regulation of cold-responsive genes in the mutants, we hypothesized that the proteins encoded by these genes might also accumulate at relatively high levels in these lines. Previous proteomic analyses of Col-0 and the autophagy mutants *atg1abct*, *atg11*, and *atg5* revealed elevated COR47 expression in the mutant background [52, 53]. To further investigate the stability of COR47 in the *atg13ab* mutant, MBP-COR47 was purified in vitro, and its stability was examined with cell-free analysis. The results revealed that the degradation rate of MBP-COR47 was inhibited in the mutant extract (Fig. 9A, B). Further expression analysis of the native promoter-driven COR47-GFP fusion protein in both Col-0 and *atg13ab* mutants revealed two specific immunoreactive sizes (~ 55 kDa and ~ 86 kDa) for COR47-GFP (Supplementary Fig. 2). At 22 °C, the COR47 protein was expressed at low levels in the wild-type plants but was elevated in the *atg13ab* mutant (Fig. 9C, D). Following prolonged cold treatment, induction of COR47 expression was observed in Col-0, while the *atg13ab* mutant exhibited even greater accumulation (Fig. 9E, F). Fluorescence microscopy of seedlings further corroborated this differential accumulation pattern (Supplementary Fig. 3).

These results suggest the constitutive accumulation of COR47 in the *atg13ab* mutant, suggesting that COR47 may be a potential substrate of autophagy. To further investigate the relationship between COR47 and autophagy, we first analyzed its subcellular localization and found that COR47 primarily localizes in the cytoplasm rather than the nucleus (Supplementary Fig. 4) as previously described [54]. However, neither yeast two-hybrid nor BiFC assays detected any interaction between COR47 and core autophagy-related proteins, although COR47 has been shown to form homodimers (Supplementary Fig. 5). Therefore, COR47 likely regulates its stability through mechanisms independent of direct interactions with core autophagy proteins.

## Discussion

Autophagy, as a protective strategy, has been demonstrated to play a positive role in plant responses to multiple stresses [55, 56]. However, in this study, we revealed a previously unrecognized negative regulatory role of autophagy in *Arabidopsis* freezing tolerance independent of cold acclimation (Fig. 1). To elucidate the mechanism underlying the enhanced cold tolerance of the autophagy-deficient plants, we analyzed protective metabolites, including proline and anthocyanins. Notably, autophagy mutants accumulated higher proline levels than wild-type plants following cold treatment, in both NA and CA conditions (Fig. 1F), whereas anthocyanin accumulation was significantly lower in these mutants (Fig. 2). These findings suggest that anthocyanins likely do not directly contribute to the cold-tolerant phenotype observed in autophagy mutants. Unlike a previous study attributing reduced anthocyanin levels in autophagy mutants to impaired vacuolar trafficking [57], our transcriptomic and qRT-PCR data demonstrated that anthocyanin biosynthesis-related genes (e.g., *DFR*, *ANS*, *CHS*) were constitutively downregulated in the *atg* mutants under basal conditions (Fig. 2 and 6), which is consistent with previous observations showing downregulation of the anthocyanin-related genes in the autophagy mutant *atg5* [58]. These results imply that autophagy may suppress anthocyanin production by potentially suppressing the flavonoid biosynthetic pathway rather than affecting vacuolar transport. Nevertheless, the molecular mechanisms by which autophagy downregulates flavonoid metabolism remain unclear and warrant further investigation.

To better understand the mechanistic contributions of autophagy to cold stress responses in *Arabidopsis*, we examined the effects of cold stress on autophagy and observed significant suppression of autophagic activity characterized by downregulation of *ATG* gene expression (Fig. 3), reduced autophagosome formation (Fig. 4A-B), and impaired ATG8 processing and lipidation (Fig. 4C-F). These findings align with prior reports of cold-induced autophagy inhibition [34, 35] (Fig. 3 and 4). However, the precise molecular mechanisms underlying cold-induced autophagy suppression remain unclear and represent an important direction for future research. Several potential mechanisms warrant consideration: (1) Cold stress may establish repressive chromatin states at autophagy gene loci through epigenetic reprogramming (e.g., H3K27me3 deposition), as suggested by dynamic epigenetic modifications of autophagy genes in *Arabidopsis* [59]; (2) in *J. curcas* seedlings, cold treatment markedly increased the phosphorylation levels of ATG13 [60], suggesting that unidentified cold-activated kinases may phosphorylate autophagic components, altering their stability or function to suppress autophagic flux. Future studies should employ integrated approaches (e.g., ChIP-qPCR, phosphoproteomics) to dissect the precise mechanisms of cold-mediated autophagic suppression.

To elucidate the broad role of autophagy in plant cold tolerance, we conducted comparative transcriptome analysis of wild-type *Arabidopsis* and autophagy-deficient *atg13ab* mutants. We observed that even under nonstress conditions, Col-0 and *atg13ab* already exhibited substantial differences in gene expression, including the expression of cold-responsive genes such as *CBFs*, *DDF1*, *B1L*, and *ZAT12* (Fig. 6). These results are consistent with a previous study showing constitutive upregulation of cold-responsive genes *CBF1*, *CBF2*, *MYB15*, and *ZAT12* in the *atg5* mutant at 60 days after sowing (60 DAS) compared to wild-type plants [58]. This may suggest that autophagy deficiency places cells in a persistent state of stress, leading to constitutive activation of the CBF/DREB1 signaling pathway even in the absence of external cold stress. The pre-activation of CBF signaling in autophagy mutants may mimic the “cold priming” phenomenon [61], potentially enabling rapid deployment of cryoprotective mechanisms without prior cold exposure. However, the precise mechanism by which these genes are activated in the mutants remains unclear. One possible explanation is that certain positive regulators of the cold response, such as transcription factors (e.g., ICE1) or kinases (e.g., OST1), may serve as autophagy substrates. However, the absence of proteomic validation in our mutants limits this interpretation. Notably, we observed cold-induced up-regulation of salicylic acid (SA)-related and fungal defense-related genes in Col-0 (Supplementary Fig. 1A), which is consistent with previous reports showing that cold activates immunity-related pathways [62]. This response pattern stems from cold stress triggers a defense-like response similar to pathogen invasion, which typically leads to elevated SA levels and the activation of defense-related genes to prepare plants for sensitization to future pathogen infection [63]. However, genes associated with immunity against bacteria, fungi, and oomycetes were downregulated before and after cold treatment in *atg13ab* mutant (Fig. 7). Given the well-studied energy-intensive nature of immune responses [64], we propose that ATG13 constitutively suppresses immune pathway activation under non-pathogenic conditions to prevent energetically costly autoimmunity, thereby redirecting limited resources toward cryoprotectant biosynthesis and CBF/DREB1 signaling pathway maintenance, ultimately conferring enhanced cold adaptability in the mutant.

Dehydrins play crucial roles in plant responses to water deficit and are considered to contribute to the protection of fragile organellar structures under adverse conditions [65, 66]. Although drought and freezing are distinct stresses, freezing-induced cellular dehydration triggers substantial accumulation of dehydrins [67]. Through cell-free and in planta expression assays, we found that the COR47 protein was strongly induced by cold in wild-type plants, whereas it exhibited sustained accumulation in autophagy mutants even under normal temperatures (Fig. 9 and Supplementary Fig. 3). This aberrant protein stabilization may account for the enhanced freezing tolerance phenotype observed in the *atg13ab* mutant. A comparative proteomic analysis of wild-type and *atg1abct* mutants under normal conditions revealed significant enrichment of cold-regulated differentially expressed proteins (DEPs), including COR47, RESPONSIVE TO DESICCATION 29A (RD29A), and LOW TEMPERATURE-INDUCED 30 (LTI30), in the *atg1abct* mutant [52]. Furthermore, studies in *atg11* and *atg5* mutants demonstrated that COR47 displayed higher protein abundance and lower degradation rates than the wild type [53]. These observations suggest a potential association between the dehydrin COR47 and autophagy, as previously described [68]. However, we did not detect any direct interaction between COR47 and core autophagy proteins through Y2H and BIFC (Supplementary Fig. 5). This finding implies the possible existence of a specific autophagy receptor that targets COR47 as a cargo for degradation via the autophagy pathway. Additionally, we observed two distinct molecular weight species of the COR47-GFP fusion protein in planta (Fig. 9 and Supplementary Fig. 2). Previous studies have demonstrated that *Arabidopsis* COR47 can form homodimers or heterodimeric complexes with dehydrins ERD10 and RAB18 [54]. However, the presence of higher molecular weight species remains challenging to explain, as such oligomeric complexes would dissociate under denaturing conditions during immunoblot analysis. Due to the lack of a COR47-specific antibody, further investigations such as mass spectrometry or native gel electrophoresis are required to determine the authentic size and oligomeric state of endogenous COR47 protein in planta.

Although we have uncovered that autophagy negatively regulates freezing stress tolerance in *Arabidopsis*, this finding contrasts with the well-established protective role of autophagy in cold resistance among cold-sensitive crops such as tomato, pepper, or eggplant [28–31, 33]. We note that studies on cold stress in these temperate plants typically use chilling treatments at ≥ 4 °C, rather than sub-zero freezing treatments. For cold-sensitive plants, 4 °C may already represent a lethal stress temperature, while for *Arabidopsis*, it serves as a preparatory temperature for cold adaptation. This difference in experimental conditions, combined with distinct cold resistance strategies in these plants, likely explains differences in autophagy function. As a cold-tolerant plant, *Arabidopsis* has evolved efficient freeze-protection mechanisms (e.g., COR protein-mediated membrane protection) [69]. This reduces its reliance on autophagy for protection and may instead suppress autophagy to prevent energy depletion. In contrast, tropical/subtropical plants lack robust freeze-protection systems [70] and may depend more on autophagy to remove cold-damaged proteins and maintain basic functions. Since this study is limited to the model plant *Arabidopsis*, whether autophagy negatively regulates freezing damage in other cold-tolerant crops requires further investigation. Moreover, our experiments involved only *Arabidopsis* seedlings, and the role of autophagy in freeze tolerance regulation throughout its entire life cycle remains unclear. Additionally, our chilling treatment lasted merely 45 days, and the performance of autophagy mutants under prolonged low-temperature conditions remains to be determined.

In summary, this study demonstrates that autophagy acts as a negative regulator of freezing tolerance in *Arabidopsis*, contrasting sharply with its well-established protective function against other abiotic stresses. This functional divergence indicates that autophagy may exert either beneficial or detrimental effects depending on the specific stress context. Further research is required to elucidate the underlying mechanisms governing this stress-dependent functional shift.

## Materials and methods

### Plant materials and growth conditions

All materials used in this work were in the *A. thaliana* accession Columbia (Col-0) background. Mutants of *atg5-1* (SAIL_129_B07) and *atg7-3* (SAIL_11_H07) were obtained from the *Arabidopsis* Biological Resource Center (ABRC). *atg13ab* (SALK_044831) and *atg1abct* were obtained from Prof. Fa Qiang Li [19, 37]. *pATG8a::GFP-ATG8a*/Col-0 transgenic line was obtained from Prof. Shi Xiao [42]. Other stable expression transgenic plants were generated in this study.

*Arabidopsis* seedlings were surface sterilized and vernalized at 4 °C for 2–3 days, then germinated on 1/2 MS medium at 22 °C under an LD photoperiod (16 h light/8 h darkness) with illumination at ~ 100 µmol m^−2^ s^−1^. After 1 week, the seedlings were transferred to soil for further growth. *N. benthamiana* was subsequently grown under LD conditions. One-month-old tobacco plants were used for transient expression assays.

### Plasmid construction and transgenic plant generation

The gene-specific primers are listed in Supplementary Table 1. To generate *proATG8a::GUS* and* proATG13a::GUS*, the *ATG8a* promoter (− 1199 to + 1) and *ATG13a* promoter (− 2000 to + 1) were amplified and inserted into pMDC163 between PacI and XbaI via homologous recombination using the ClonExpress II One Step Cloning Kit (Vazyme, Nanjing, China). For *proCOR47::COR47-GFP*, a 2000 bp promoter region of *COR47* (− 2000 to + 1) was PCR-amplified and inserted into pCAMBIA1305 (c-eYFP) between SacI and BamHI. A 942 bp genomic fragment of *COR47* was then fused upstream of GFP using the ClonExpress II One Step Cloning Kit. All constructs were sequence-verified before transformation into *A. tumefacien*s (GV3101) for floral dip. Primary transformants were selected by antibiotic resistance and confirmed by PCR.

### Assays for freezing tolerance, chlorophyll content, electrolyte leakage, and proline content

Freezing tolerance was assessed as previously described [71]. Briefly, 14-day-old plants grown at 22 °C on 1/2 MS plates were treated with or without cold acclimation (4 °C for 2 d) and then subjected to a freezing assay. The program was set at 0 °C, and 1 °C h was dropped to the desired temperature. After freezing treatment, the plants were incubated at 4 °C in the dark for 12 h and then transferred to 22 °C for an additional 2–3 d. Chlorophyll was extracted and measured as described [72]. Electrolyte leakage assays were performed as described [71]. The proline content was measured as previously described [45]. At least three independent experiments were performed, and each experiment was performed with three technical replicates.

### Chilling stress response assay and anthocyanin extraction

For chilling stress, 5-day-old wild-type, *atg1abct*, *atg13ab*, *atg7-3*, and *atg5-1* seedlings grown on 1/2 MS plates at 22 °C were transferred to a 4 °C growth chamber (16 h light/8 h darkness with illumination at ~ 100 µmol m^−2 s−1^) and maintained for the indicated times. At least three independent experiments were performed, and each experiment was performed with three technical replicates.

Anthocyanin measurement was performed as described previously [41]. *Arabidopsis* seedlings were incubated in extraction buffer (methanol containing 1% HCl) overnight at 4 °C in the dark. The samples were centrifuged, and the supernatants were collected for absorbance quantification at 530 and 657 nm. (A_530_ − 0.25 × A_657_) per gram fresh weight was used to quantify the relative amounts of anthocyanins.

### qRT-PCR analysis

RNA extraction was performed according to the instructions of the total RNA kit (OMEGA). 5 × PrimeScript™ RT Master Mix (TAKARA) was used to synthesize cDNA. qRT-PCR analysis was performed using SYBR Premix Ex Taq II (TaKaRa) and a StepOne PCR instrument. The 2^−△△CT^ method was used to calculate the relative expression of genes. *Ubiquitin 10* (*UBQ10*) was used as a reference gene. The gene-specific primers used for qRT-PCR are listed in Supplemental Table 1.

### GUS staining and quantitative GUS activity assay

Transgenic plants harboring the *proATG13a::GUS* or *proATG8a::GUS* constructs were immediately immersed in GUS staining solution (5 mM EDTA disodium salt, 50 mM Na_2_HPO_4_ buffer, 2 mM K_4_Fe(CN)_6_, 2 mM K_3_Fe(CN)_6_, 0.1% Triton X-100, 0.04 mg/mL X-gluc (B5285, Sigma), pH 7.0) after various durations of 4 °C treatment, incubated for 5 h at 37 °C, and then decolorized with 70% ethanol. The stained tissues were observed and photographed with a Zeiss Discover.v20 imaging system.

For the β- GUS activity assay, the enzymatic activity was determined based on the hydrolysis of p-nitrophenyl-β-D-glucuronide (pNPG) to release p-nitrophenol (pNP), which was quantified by monitoring the increase in absorbance at 405 nm. Briefly, 0.1 g of cold-treated seedlings were homogenized in 1 mL of extraction buffer on ice, followed by centrifugation at 15,000 rpm, 4 °C for 10 min. The supernatant was collected as the crude enzyme extract. According to the manufacturer’s instructions (Grace Biotechnology, Cat. No. G0579F), the reaction mixture was incubated at 37 °C for 30 min, and the absorbance of the supernatant was measured at 405 nm. The β-GUS activity (μmol/h/g FW) was calculated by 3.71 × (A_405_ + 0.0037)/W × D. W, sample fresh weight (g); D, dilution factor.

### Drug treatment and confocal laser scanning microscopy

For concanavalin A (ConA) treatment, seedlings of *proATG8a::GFP-ATG8a*/Col-0 were first grown on 1/2 MS agar media for 5 days at 22 °C. The sterile seedlings were then transferred to 1/2 MS liquid media and either continued culturing at 22 °C for 12 h (control) or exposed to 4 °C in a growth chamber for 12 h under a standard photoperiod (16 h light/8 h dark cycle with ~ 100 µmol m⁻^2^ s⁻^1^ light intensity). After treatment, the seedlings were allowed to recover for 6 h at the normal temperature (22 °C). Subsequently, either DMSO (control) or 0.5 μM ConA was added to the liquid medium, followed by further incubation at 22 °C for 4 h. Following incubation, the GFP labeled autophagosomes in the roots were visualized with a Nikon A1 + confocal laser scanning microscopeusing 40 × water objectives. Excitation was performed at 488 nm, emission was collected at 500–530 nm. Three to four representative images in the root elongation zone were photographed per seedling, and the number of GFP-ATG8a puncta in each image was counted and averaged. A total of ten seedlings were observed per treatment.

### Protein isolation and immunoblot analysis

For protein extraction, *Arabidopsis* samples were ground and homogenized in ice-cold extraction buffer (10 mM HEPES, pH 7.5; 100 mM NaCl; 1 mM EDTA pH 8.0; 10% Glycerol; 0.5% Triton X-100; 1 × cocktail). Samples were incubated on ice for 10–15 min and centrifuged at 4 °C for 10 min at 12,000 g. The supernatant was used for electrophoresis. For immunoblot analysis, total proteins were subjected to SDS-PAGE and electrophoretically transferred to a polyvinylidene fluoride membrane (Immobilon-P; Millipore). For the ATG8a assay, total proteins were separated on 15% SDS-PAGE gels in the presence of 6 M urea. The antibodies used for protein blot analysis were against ATG8a (Abcam, ab77003, 1:1000), GFP (Abmart, M20004, 1:5000), and β-actin (CWBIO, CW0264, 1:5000).

### High-throughput mRNA sequencing analysis

Fourteen-day-old seedlings grown on 1/2 MS medium at 22 °C were treated at 4 °C for 0, 3 or 24 h. Total RNA was extracted. and 3 μg of RNA for each sample was used for library construction and subsequent RNA-deep sequencing on the Illumina HiSeq 2500 platform. RNA-Seq data were collected from two independent experiments. The adaptor sequences and low-quality sequence reads were removed from the data sets. Raw sequences were transformed into clean reads after data processing. These clean reads were then mapped to the reference genome sequence. Only reads with a perfect match or one mismatch were further analyzed and annotated on the basis of the reference genome. HISAT2 tools were used for mapping with the reference genome. Gene function was annotated on the basis of TAIR10. Differential expression analysis of two conditions/groups was performed via the DESeq2 R package (1.26.0). DESeq2 provides statistical routines for determining differential expression in digital gene expression data via a model based on the negative binomial distribution. The resulting* P* values were adjusted via Benjamini and Hochberg’s approach for controlling the false discovery rate. Genes with FDR < 0.05 &|log2(foldchange)|≥ 1 found by DESeq2 were considered differentially expressed. Gene Ontology (GO) enrichment analysis of the differentially expressed genes (DEGs) was implemented via the GOseq R package-based Wallenius non-central hyper-geometric distribution, which can adjust for gene length bias in DEGs [73].

#### Recombinant protein expression and cell-free assay

For the expression of recombinant protein in prokaryotic cells, the CDSs of *COR47* were cloned and inserted into the pMal-cRi (MBP tag) vector and transformed into the *E. coli* strain Rosetta, MBP tagged proteins were purified using PurKine™ MBP-Tag Dextrin Resin 6FF (BMR20206, Abbkine) following the manufacturer’s instructions.

For the cell-free assay, 200 − 400 mg of 10-day-old normally growing plants was collected, and total protein was extracted via protein degradation buffer (50 mM Tris–HCl pH 7.5, 100 mM NaCl, 5 mM DTT, and 1 mM ATP). Add 100 ng of purified MBP-COR47 protein, and the mixture was incubated at room temperature for 0, 15, 30, 45, or 60 min. Equal amounts of the reaction mixture were collected at each time point to detect MBP-COR47 expression using anti-MBP (Abmart, 15089-1-AP, 1:5000).

#### Transient expression in tobacco epidermal cells and *Arabidopsis* protoplasts

For transient expression of tobacco epidermal cells, the resulting plasmids were introduced into *A. tumefaciens* strain GV3101, overnight cultures of which were resuspended in 5 mL infiltration buffer (0.15 M acetosyringone dilute in DMSO; 0.01 M MES, pH 7.5; 0.01 M MgCl_2_), incubated at room temperature for 4 h in the darkness, and then used for direct infiltration of 4 to 6-week-old *N. benthamiana* leaves. Leaf sections of approximately 2 mm × 2 mm were excised 36–48 h after infiltration were visualized with fluorescence microscope (Leica DMi8).

Transient expression assays in *Arabidopsis* protoplasts were conducted essentially as described previously [74, 75]. Briefly, healthy plants (3–4 weeks old, short-day grown) are used for protoplast isolation. Fully expanded rosette leaves are harvested and sliced. The leaves are then submerged in an ice-chilled enzyme solution (0.015 g/mL cellulase R10, 0.004 g/mL macerozyme R10, 0.4 M mannitol, 20 mM KCl, 20 mM MES pH 5.7, 10 mM CaCl₂, 5 mM β-mercaptoethanol, and 0.1% BSA). After vacuum infiltration for 30 min, the mixture is incubated in the dark at room temperature with gentle shaking (40–60 rpm) for 3–4 h. The digested protoplast suspension is filtered and centrifuged (100 × *g*, 2–3 min, 4 °C), and washed twice with cold W5 solution (154 mM NaCl, 125 mM CaCl₂, 5 mM KCl, 5 mM glucose, 2 mM MES, pH 5.7). Protoplasts are counted and resuspended in MMg solution (0.4 M mannitol, 15 mM MgCl₂, 4 mM MES, pH 5.7) at ~ 2 × 10^5^ cells/ml. For transformation, 100 µL of protoplast suspension is mixed with 100 µg COR47-GFP plasmid, followed by an equal volume of PEG solution (40% PEG 4000, 0.2 M mannitol, 100 mM CaCl₂). After 5–30 min incubation at room temperature, the reaction is stopped by adding 4–5 volumes of W5 solution. The transformed protoplasts are pelleted (100 ×* g*, 2 min), resuspended in W1 solution (0.5 M mannitol, 20 mM KCl, 4 mM MES, pH 5.7), and incubated at 22 °C for 16 h to allow sufficient expression of the fusion protein. Subcellular localization was subsequently examined using a fluorescence microscope (Leica DMi8).

#### Y2H and BiFC

For yeast two-hybrid (Y2H) assay, pairwise AD and BD were co-transformed into yeast strain Y2H gold (Clontech). Cells transformed with both plasmids were selected for growth 2 d at 30 °C on a synthetic dropout medium lacking leucine and tryptophan. Transformants were selected on synthetic defined (SD) medium lacking leucine and tryptophan (-Leu/-Trp) and incubated at 30 °C for 48 h. Protein–protein interactions were assessed by growth on selective SD medium deficient in leucine, tryptophan, histidine, and adenine (-Leu/-Trp/-His/-Ade) at 30 °C for 48 h. For interaction validation, single colonies were resuspended in sterile H₂O to OD600 = 0.1, and 5 µL aliquots were spotted onto both selection media followed by incubation at 30 °C for 48 h.

For the bimolecular fluorescence complementation (BiFC) assay, the recombinant plasmids were introduced into *A. tumefaciens* strain GV3101, followed by transient expression in *N.benthamiana* leaves through agroinfiltration as previously described.

## Conclusions

In this study, we revealed that unlike its well-described positive roles in common stresses such as salt, drought, and submergence, autophagy functions as a negative regulator of cold stress responses in *Arabidopsis*. The autophagy-deficient mutants exhibited enhanced freezing tolerance regardless of cold acclimation. Under chilling conditions, autophagy positively regulates anthocyanin accumulation. Cold treatment significantly suppressed autophagic activity, as evidenced by the transcriptional downregulation of autophagy-related genes, reduced autophagosome formation, and decreased ATG8 protein abundance. Notably, cold-responsive genes were constitutively upregulated in the autophagy mutants, which is consistent with the accumulation of the cold-regulated protein COR47. These findings collectively demonstrate the suppressive role of autophagy in low-temperature adaptation in *Arabidopsis*.

## Electronic supplementary material

Below is the link to the electronic supplementary material.

Supplementary Material 1

Supplementary Material 2

Supplementary Material 3

Supplementary Material 4

Supplementary Material 5

Supplementary Material 6

Supplementary Material 7

Supplementary Material 8

Supplementary Material 9

## Data Availability

All the data generated or analyzed during this study are included in this article (and its supplementary information files). The datasets supporting the conclusions of this article are included within the article (and its additional files). Sequencing database could download from NCBI under the BioProject accession number PRJNA1262711, or SRA accession number (SRR33577902, SRR33577896, SRR33577893, SRR33577899, SRR33577901, SRR33577903, SRR33577900, SRR33577897, SRR33577895, SRR33577892, SRR33577894, SRR33577898), and the data will be shared on reasonable request of the corresponding author.

## Abbreviations

ANS: Anthocyanin synthase
ATG: AuTophaGy
B1L: Bypass1-like
CBF: C-repeat binding factors
CHS: Chalcone synthase
ConA: Concanavalin A
COR47: Cold-regulated 47
DAS: Days after sowing
DDF1: Dwarf and delayed flowering 1
DEG: Differentially expressed gene
DEP: Differentially expressed protein
DFR: Dihydroflavonol 4-reductase
DREB: Dehydration-responsive element binding
GFP: Green fluorescent protein
Gols3: Galactinol synthase 3
GUS: Glucuronidas
LTI30: Low-temperature-induced 30
NBR1: Neighbor of BRCA1
PE: Phosphatidyl-ethanolamine
PI3K: Phosphatidylinositol 3-kinase
PI3P: Phosphatidylinositol 3-phosphate
RD29A: Responsive to desiccation 29a
SNARE: Soluble NSF attachment protein receptor
UPS: Ubiquitin–proteasome system
ZAT12: Zinc finger of *Arabidopsis thaliana* 12
