# Supplementary material for: Autophagy deficiency confers freezing tolerance in Arabidopsis thaliana
Source: BMC Plant Biol. 2025 Jul 30;25:994. doi: 10.1186/s12870-025-07066-9 (PMC12312434; doi:10.1186/s12870-025-07066-9)
Supplement: Supplementary file 1 — Supplementary Material 1 [file 12870_2025_7066_MOESM1_ESM.docx]

**
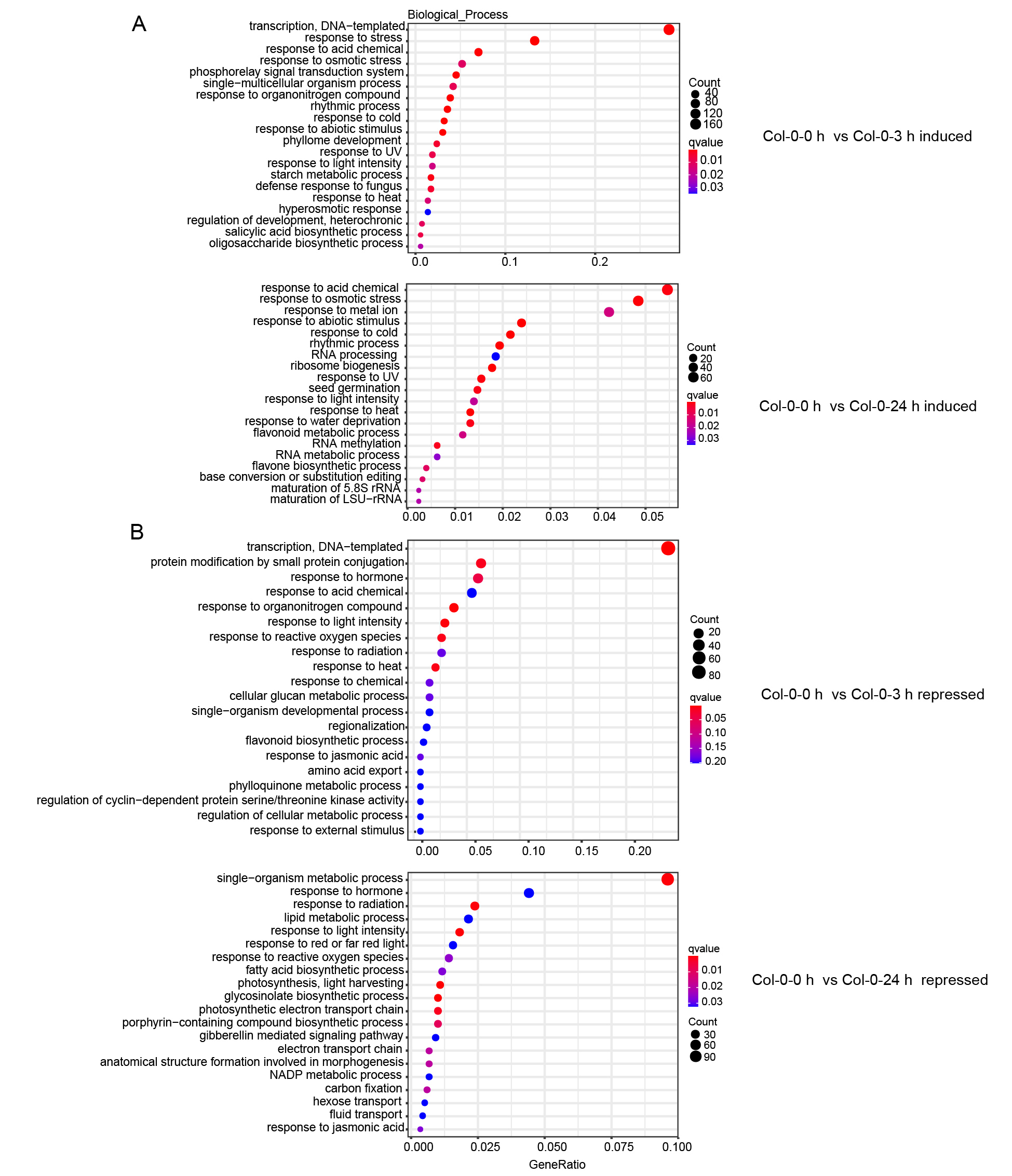
**

**Supplementary Figure 1.** GO enrichment analysis of DEGs in Col-0 before and after cold treatment at 3 h and 24 h. The enrichment analysis of up-regulated DEGs at 3 h and 24 h (A), down-regulated DEGs at 3 h and 24 h (B).

**
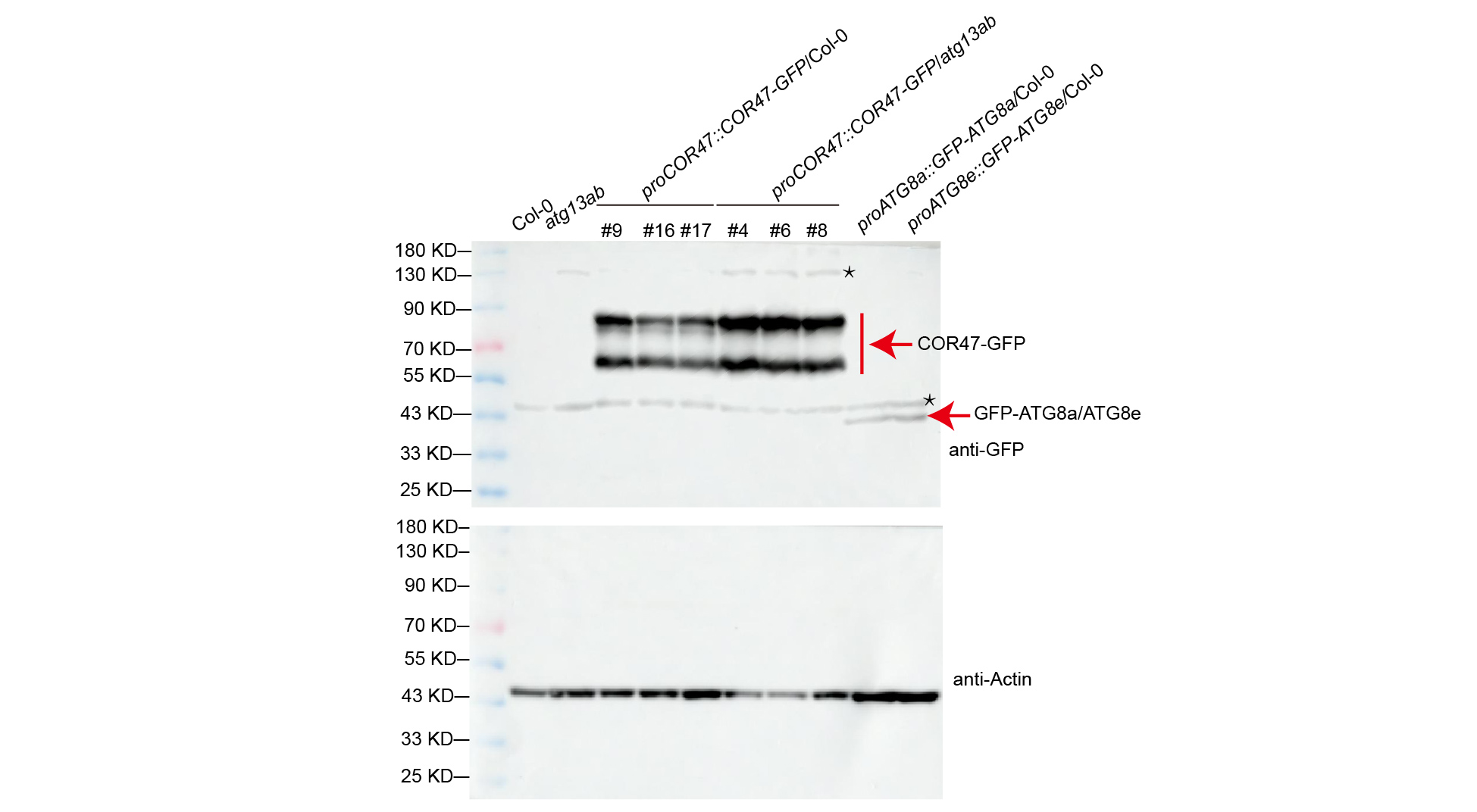
**

**Supplementary Figure 2.** Identification of stable transgenic plants expressing *proCOR47::COR47-GFP* in Col-0 and *atg13ab*. Col-0, *atg13ab*, *proATG8a::GFP-ATG8a*/Col-0, and *proATG8e::GFP-ATG8e*/Col-0 were used as controls. Black asterisks indicate non-specific bands, while red arrows indicate specific bands.

**
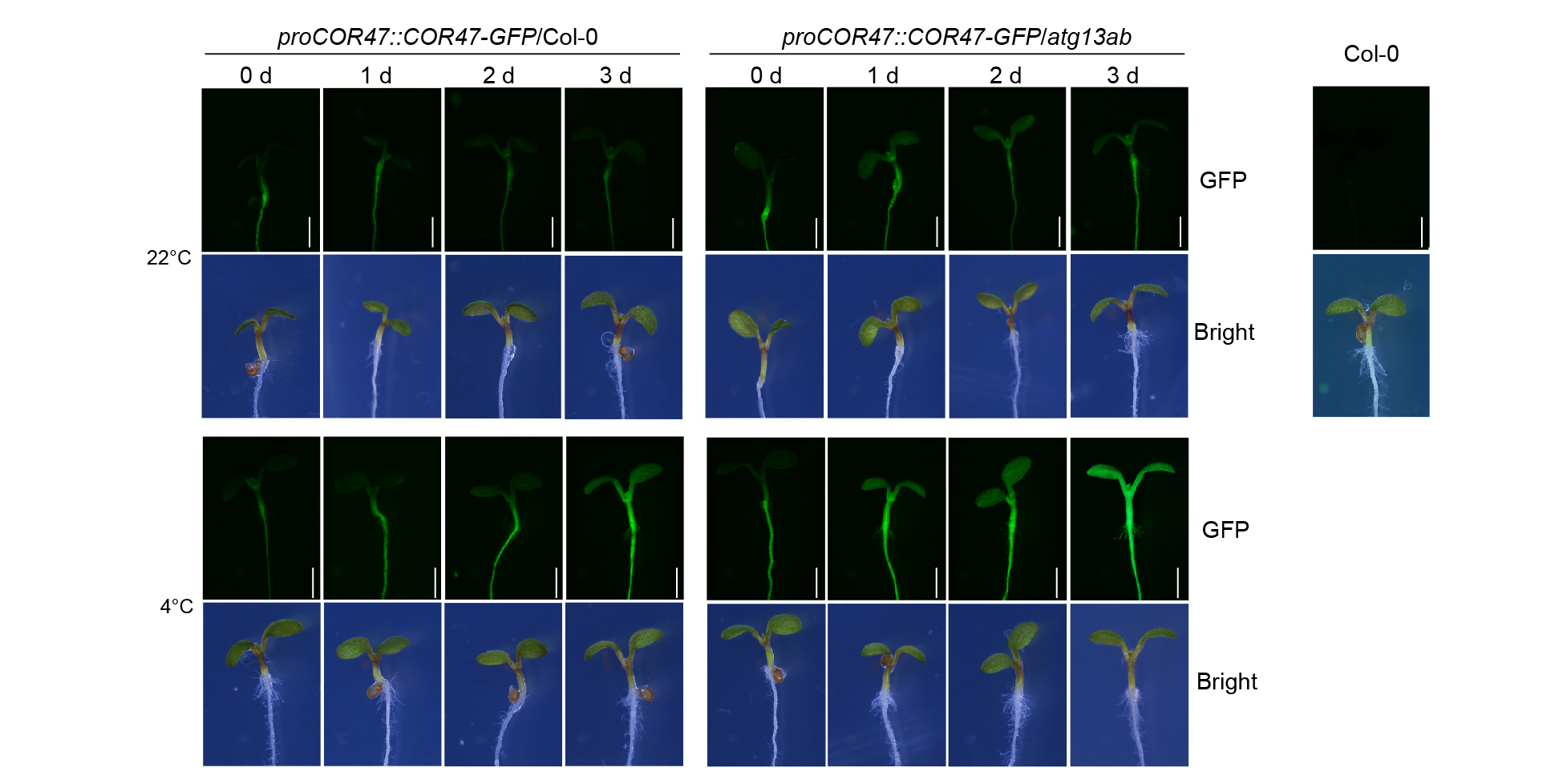
**

**Supplementary Figure 3.** Representative images of fluorescence signals of COR47-GFP in Col-0 and *atg13ab* mutants under 22°C and 4°C conditions. Scale bars, 1 mm.


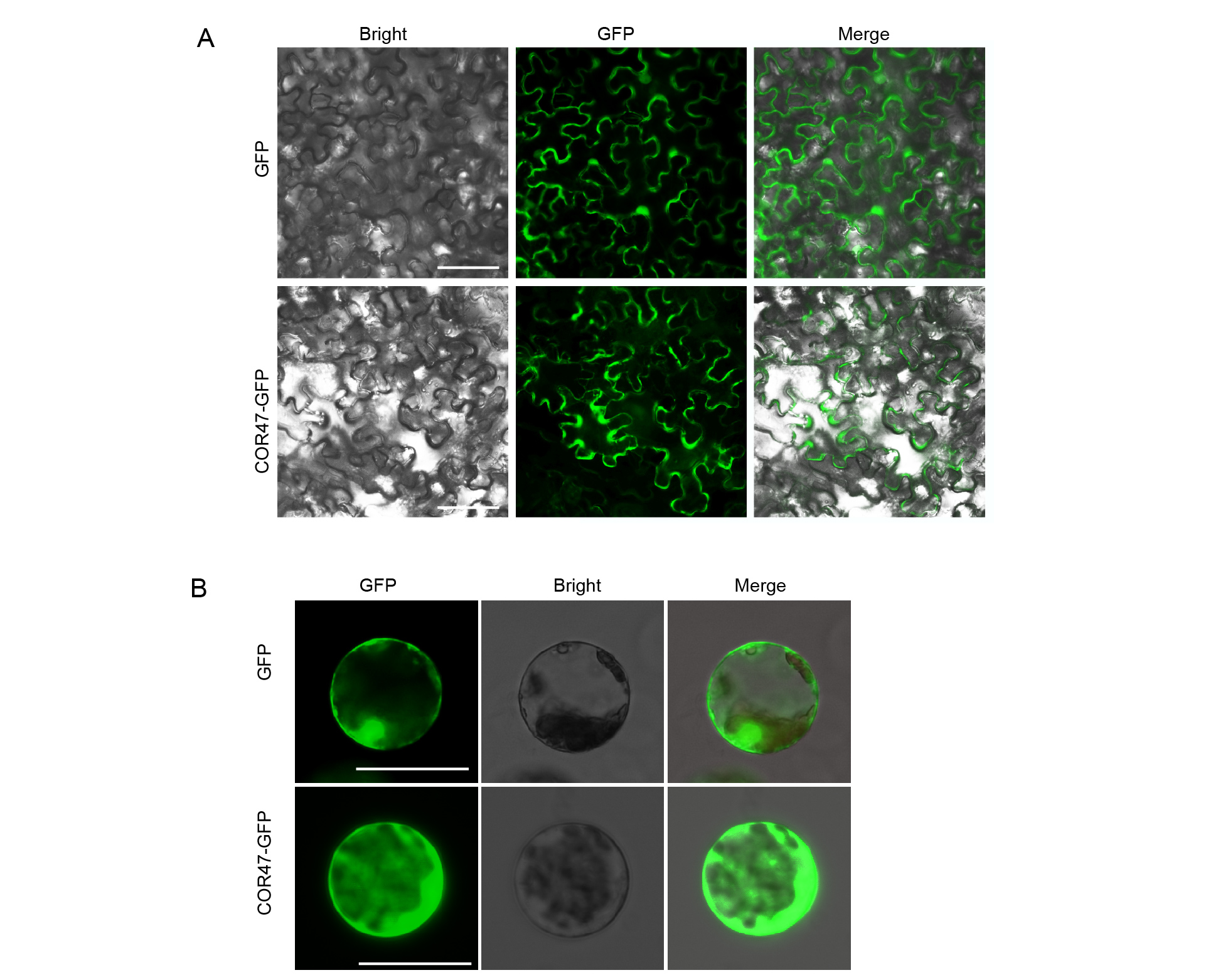


**Supplementary Figure 4.** COR47 protein is localized in the cytoplasm. **A** Subcellular localization of 35S promoter-driven free-GFP (upper panel) or COR47-GFP (lower panel) in tobacco epidermal cells. Scale bars, 40 µm. **B** Subcellular localization of free-GFP (upper panel) or COR47-GFP (lower panel) in *Arabidopsis* protoplasts. Scale bars, 50 µm.


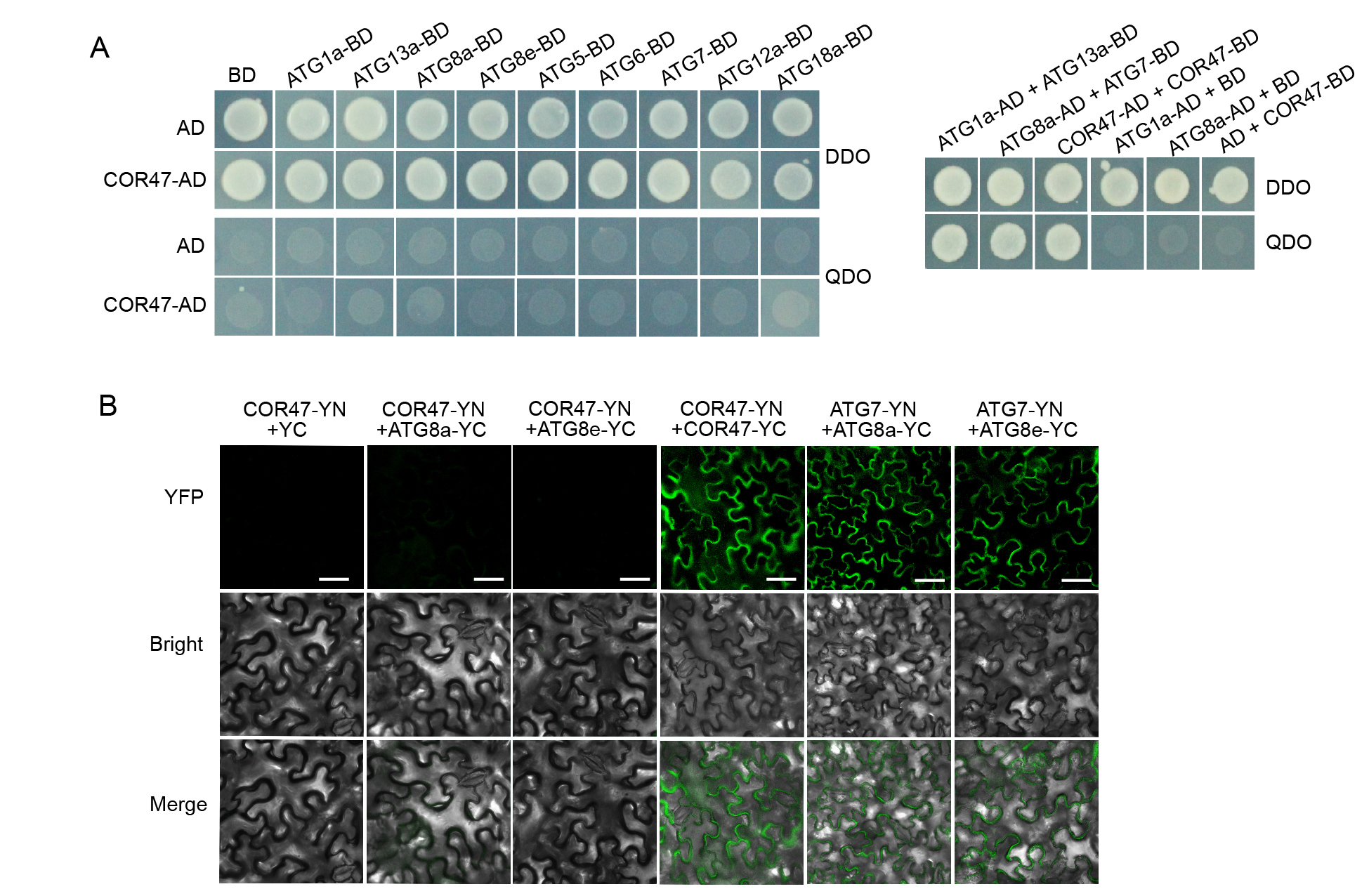


**Supplementary Figure 5.** COR47 does not directly interact with core autophagy proteins. (A) No interaction between COR47 and ATGs (ATG8a, ATG8e, ATG1a, ATG13a, ATG5, ATG6, ATG7, ATG12a, or ATG18a) in yeast two-hybrid (Y2H) assays. Paired ATG1a-AD/ATG13a-BD, ATG8a-AD/ATG7-BD, and COR47-AD/COR47-BD, were used as positive controls. DDO, SD medium lacking Trp and Leu; QDO, SD medium lacking Trp, Leu, His, and Ade. (B) No interaction between COR47 and ATG8a/ATG8e in bimolecular fluorescence complementation (BiFC) assays. Paired COR47-YN/COR47-YC, ATG7-YN/ATG8a-YC, and ATG7-YN/ATG8e-YC, were used as positive controls. Scale bars, 40 µm.
