# Supplementary material for: Autophagy deficiency confers freezing tolerance in Arabidopsis thaliana
Source: BMC Plant Biol. 2025 Jul 30;25:994. doi: 10.1186/s12870-025-07066-9 (PMC12312434; doi:10.1186/s12870-025-07066-9)
Supplement: Supplementary file 2 — Supplementary Material 2 [file 12870_2025_7066_MOESM2_ESM.docx]

**Original images of Figure 4C**

**
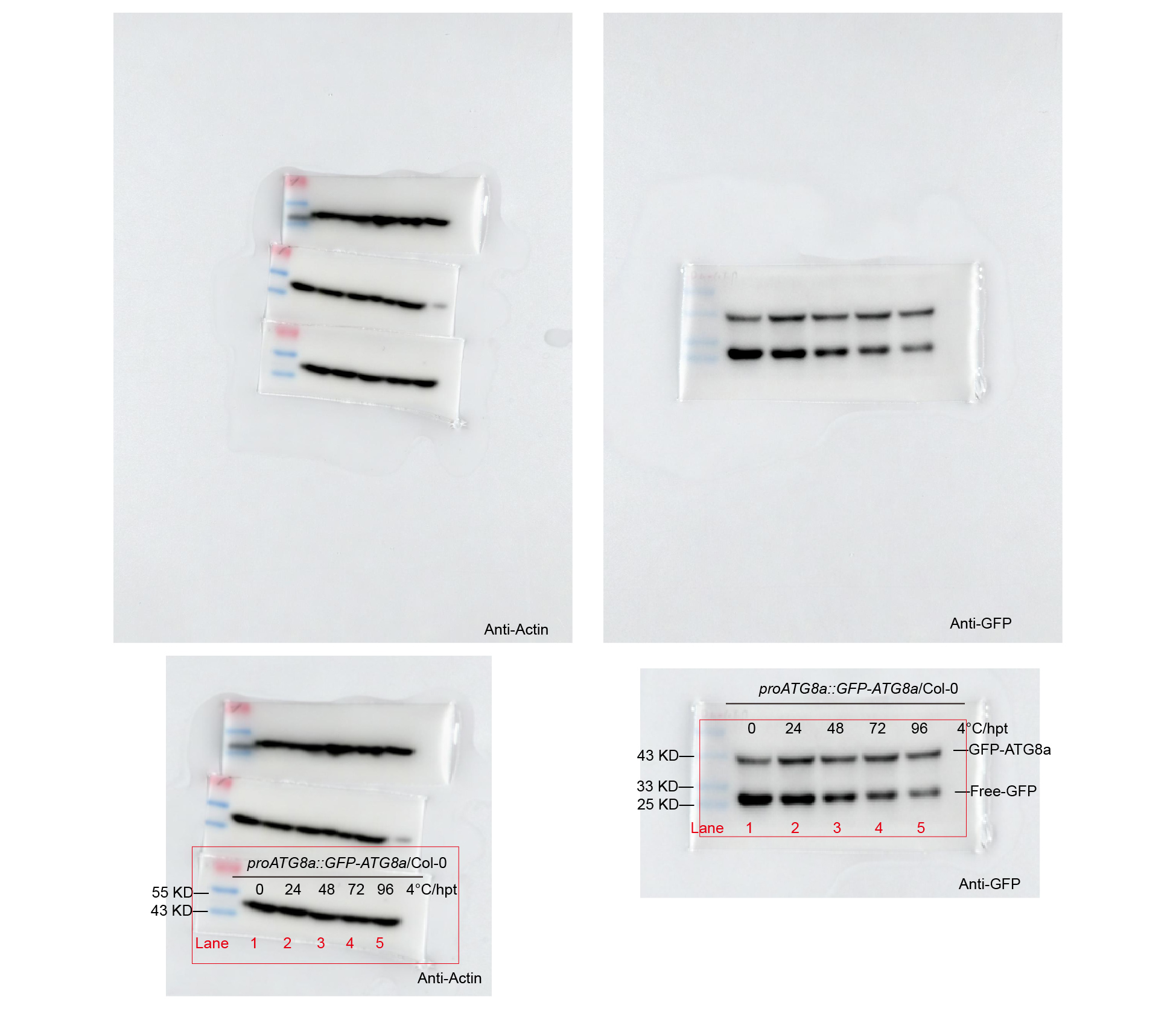
Fig. 4C** Immunoblotting analysis showing the processing of GFP-ATG8a in WT upon 4°C treatment for indicated times (Lane 1 to 5 indicates: 0, 24, 48, 72, 96 h upon 4°C treatment). Actin was used as a protein loading control. hpt, hours post-treatment.

**Original images of Figure 4E**

**
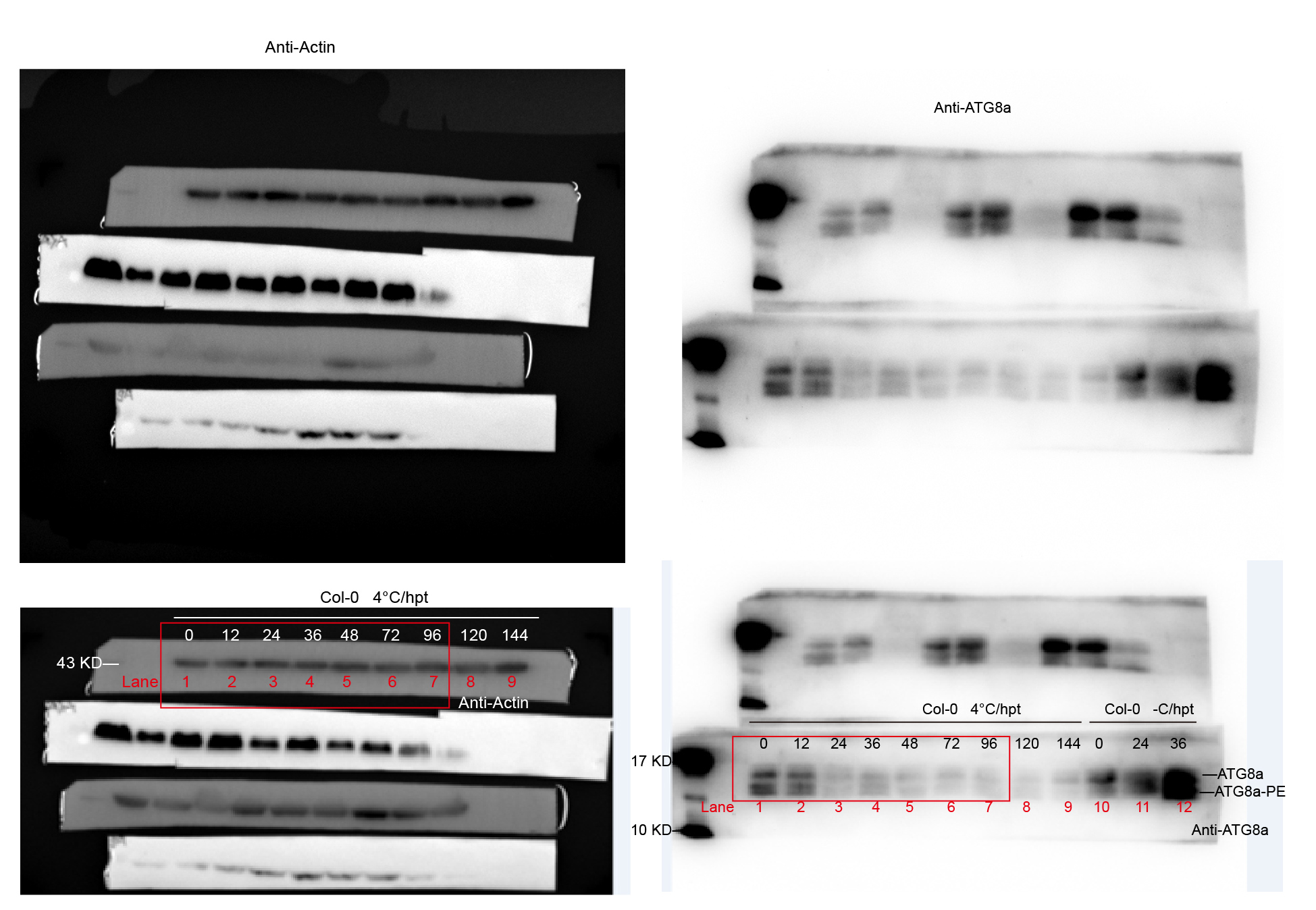
**

**Fig. 4E** Immunoblot detecting the ATG8 lipidation level in Col-0 upon 4°C treatment for indicated times (Lane 1 to 9 indicates: 0, 12, 24, 36, 48, 72, 96, 120, 144 h upon 4°C treatment). Fixed-carbon starvation treatment was set as the control group (Lane 10 to 12), since fixed-carbon starvation is the most commonly used method for autophagy induction. Actin was used as a protein loading control. hpt, hours post-treatment.

**Original images of Figure 9A**


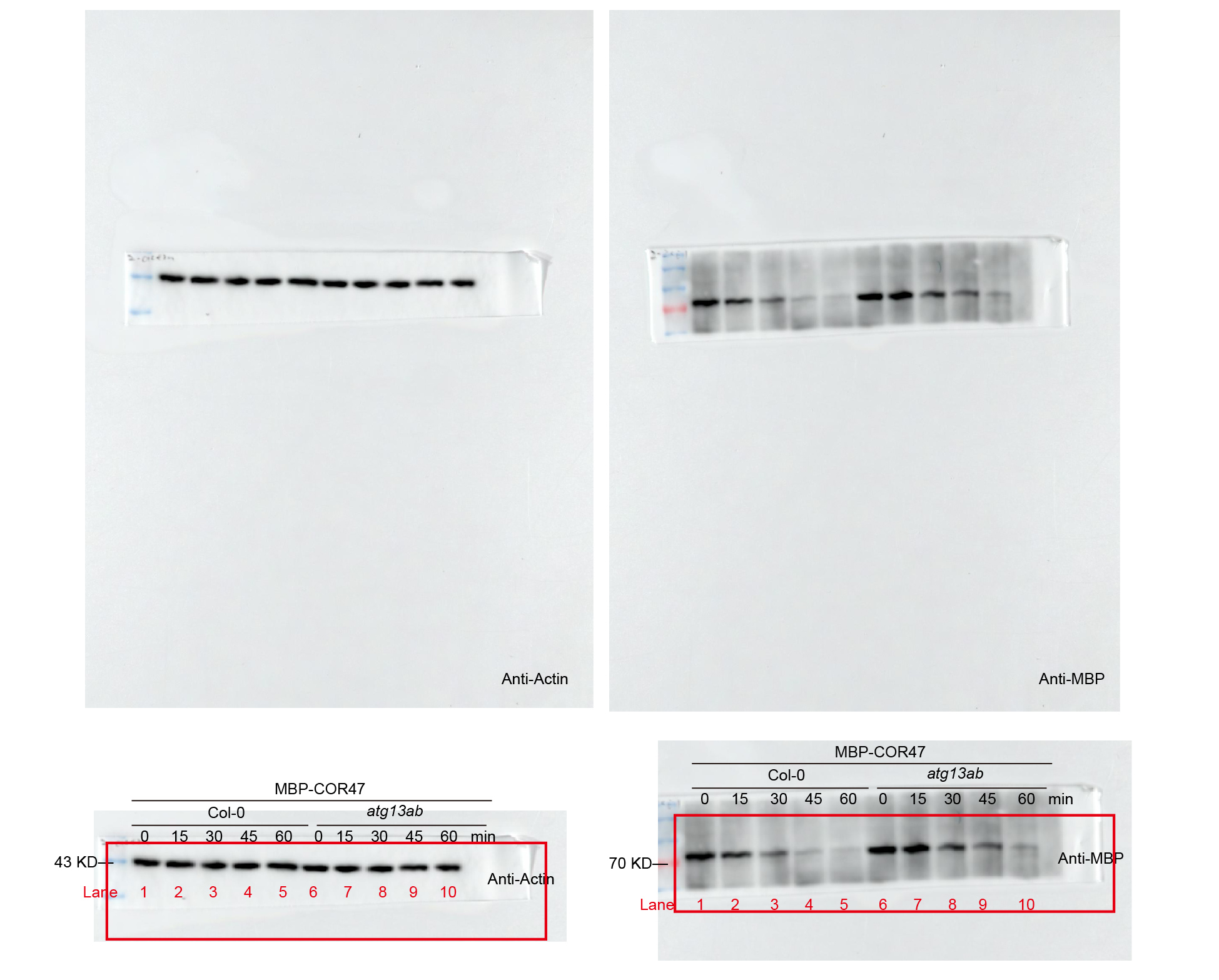
**Fig. 9A** Degradation of COR47 was inhibited in the autophagy mutant *atg13ab* in the cell-free degradation assay. Recombinant puriﬁed MBP-COR47 was incubated in equal amounts of total proteins extracted from 10-day-old Col-0 and *atg13ab* seedlings in the presence of 1 mM ATP with 5 μM MG132 for indicate times (Lane 1 to 10). MBP-COR47 was detected with anti-MBP antibody.

**Original images of Figure 9C**

**
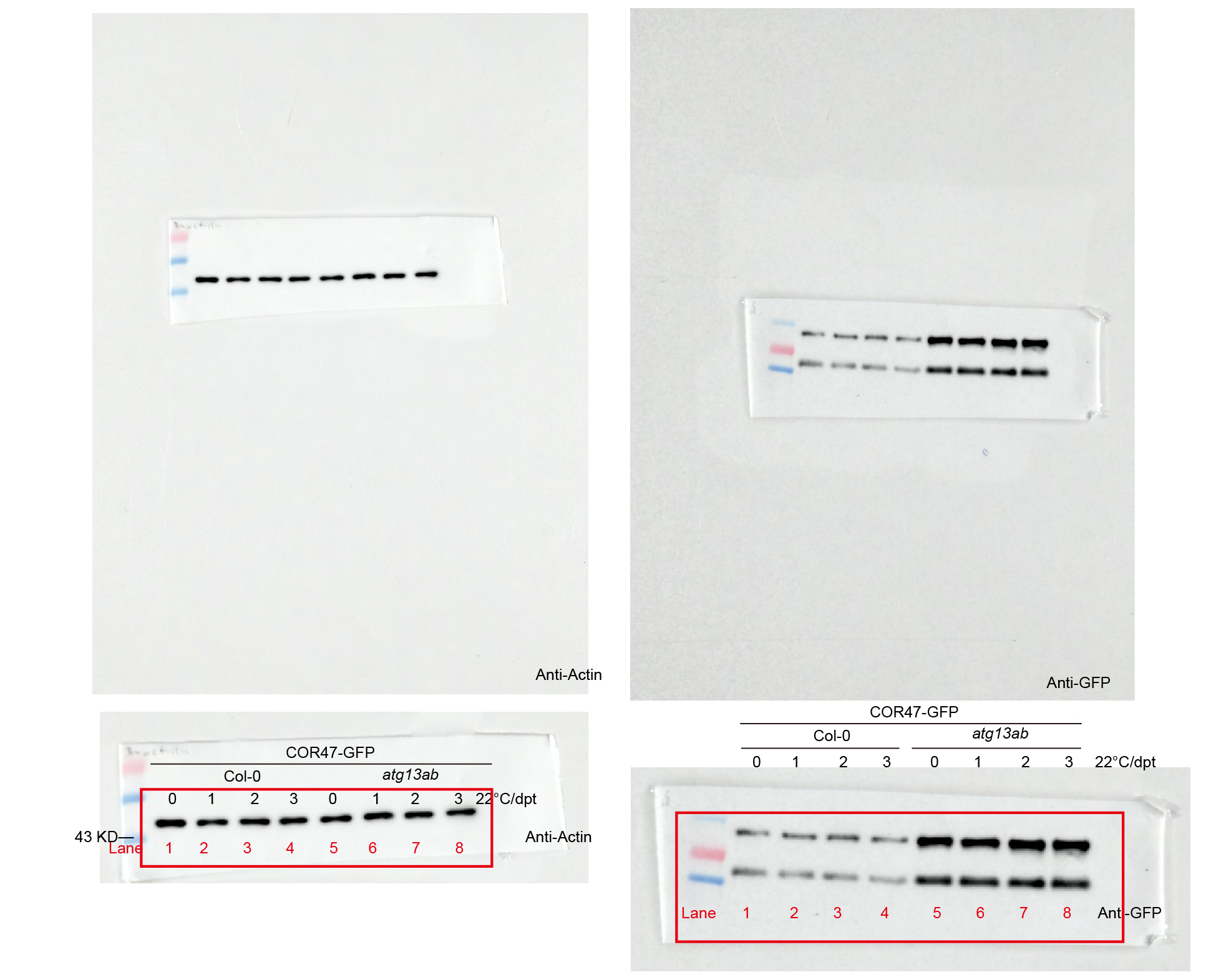
Fig. 9C** The stability of COR47-GFP in Col-0 and *atg13ab* at 22°C (Lane 1 to 8). 10-day-old Col-0 and *atg13ab* expressing *proCOR47::COR47-GFP* were maintained at 22°C. Total proteins were extracted and subjected to immunoblotting using an anti-GFP antibody.

**Original images of Figure 9E**


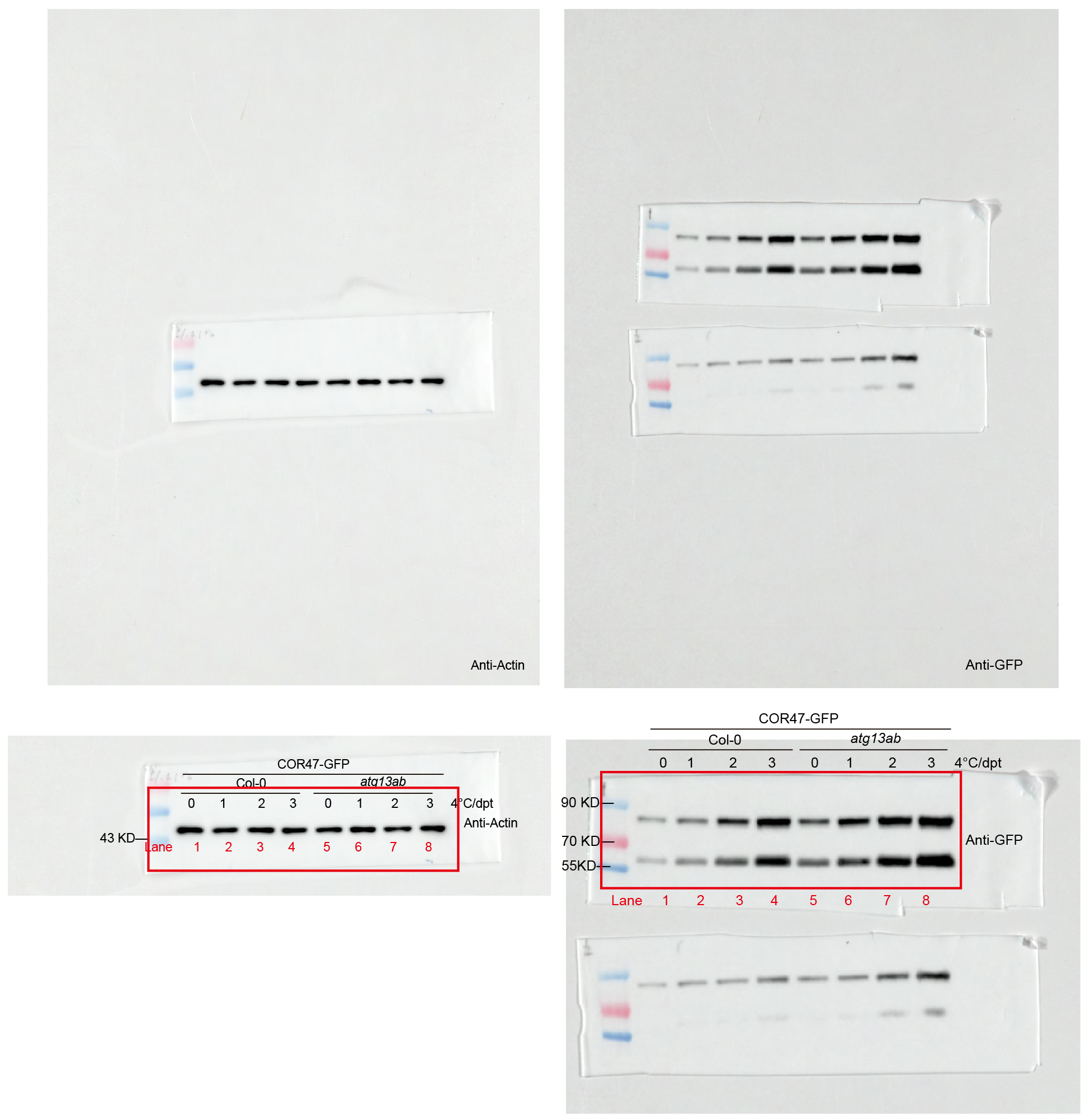
**Fig. 9E** The stability of COR47-GFP in Col-0 and *atg13ab* at 4°C. 10-day-old Col-0 and *atg13ab* expressing *proCOR47::COR47-GFP* were maintained at 4°C. Total proteins were extracted and subjected to immunoblotting using an anti-GFP antibody.

**Original images of Supplementary Figure 2**

**
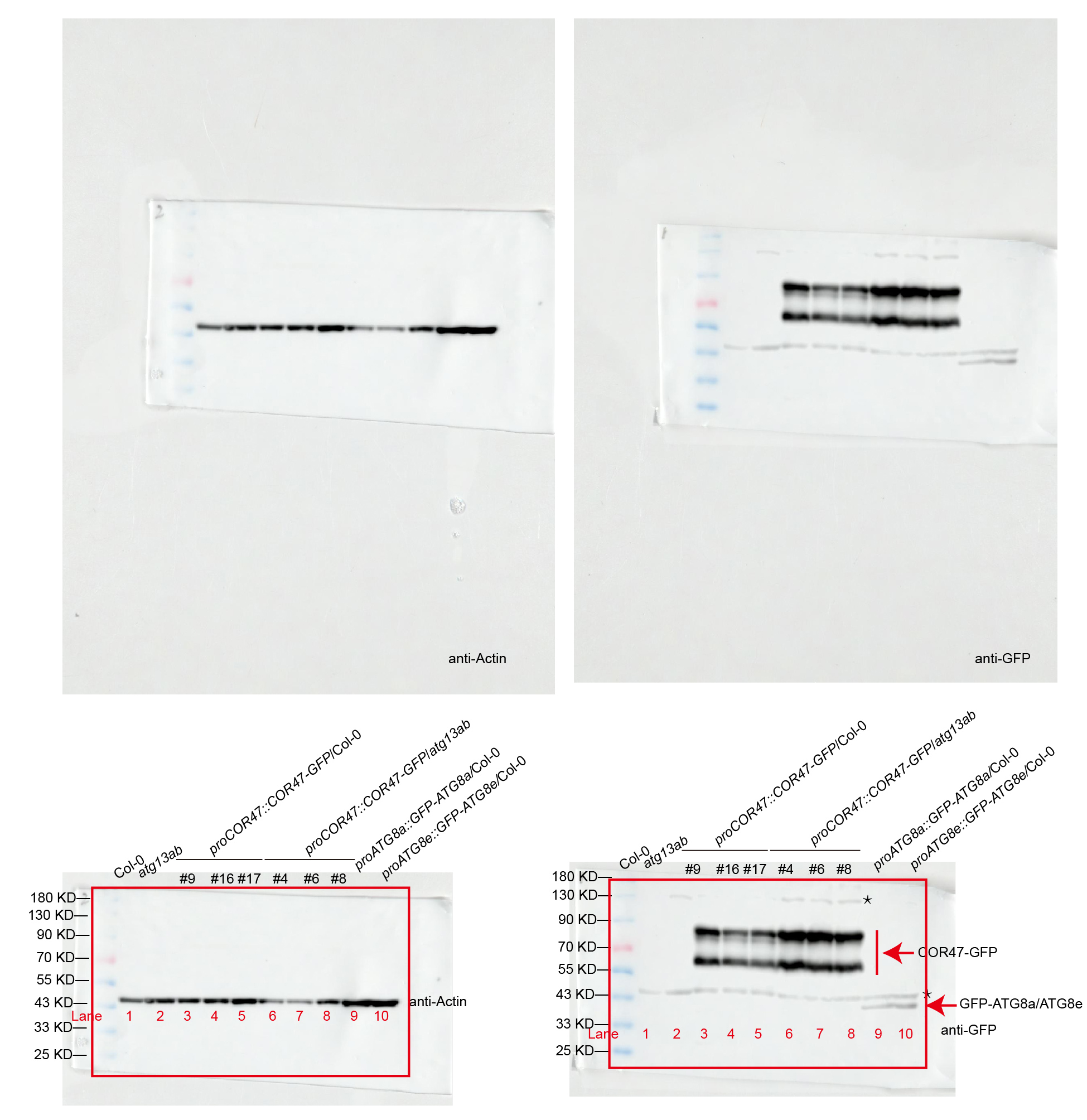
Supplementary Figure 2.** Identification of stable transgenic plants expressing *proCOR47::COR47-GFP* in Col-0 and *atg13ab* (Lane 3 to 8). Col-0, *atg13ab* (Lane 1 to 2), *proATG8a::GFP-ATG8a*/Col-0, and *proATG8e::GFP-ATG8e*/Col-0 (Lane 9 to 10) were used as controls. Black asterisks indicate non-specific bands, while red arrows indicate specific bands.
