# Supplementary material for: Autophagy deficiency confers freezing tolerance in Arabidopsis thaliana
Source: BMC Plant Biol. 2025 Jul 30;25:994. doi: 10.1186/s12870-025-07066-9 (PMC12312434; doi:10.1186/s12870-025-07066-9)
Supplement: Supplementary file 3 — Supplementary Material 3 [file 12870_2025_7066_MOESM3_ESM.docx]

**Supplementary Table 1. Primers used in this study.**

| Primer Name | Sequence (5’-3’) |
| --- | --- |
| qCBF1-F | GCATGTCTCAACTTCGCTGA |
| qCBF1-R | ATCGTCTCCTCCATGTCCAG |
| qCBF2-F | TGACGTGTCCTTATGGAGCTA |
| qCBF2-R | CTGCACTCAAAAACATTTGCA |
| qCBF3-F | GATGACGACGTATCGTTATGGA |
| qCBF3-R | TACACTCGTTTCTCAGTTTTACAAAC |
| qCOR15a-F | GCTTCAGATTTCGTGACGGATAAAAC |
| qCOR15a-R | GCAAAACATTAAAGAATGTGACGGTG |
| qCOR47-F | CAGTGTCGGAGAGTGTGGTG |
| qCOR47-R | ACAGCTGGTGAATCCTCTGC |
| qGols3-F | GGAGTGGTTGGTCTGGCTAA |
| qGols3-R | TTGGTTATCCGGTGGGTAAA |
| qDDF1-F | GTGCGGGAAGGAGAGTGTTT |
| qDDF1-R | GTCGGTTCTCTGACTTCGCA |
| qZAT12-F | TGAAAACGTCGTCGCATCCT |
| qZAT12-R | AAGCCACTCTCTTCCCACTG |
| qB1L-F | CGCGACGGTATCGAACAGAT |
| qB1L-R | AGAGCTTTCTTAGCTCGCCG |
| qCHS-F | TCAAGCGCATGTGCGACAA |
| qCHS-R | ACTTCGACCACCACGATGTC |
| qDFR-F | GCTCTCTCCTATCACTCGGAAC |
| qDFR-R | GAGCGTTGCATAAGTCGTCC |
| qANS-F | TGCAAACGATCAAGCCACTG |
| qANS-R | ACTCACTCGTTGCTTCTATGT |
| qATG5-F | ACTGATACCATGTGAAGGAG |
| qATG5-R | GTATAGGCATCAAGATCACC |
| qATG7-F | GAAGATTGTCTAGGTCGTGG |
| qATG7-R | CCTGCTTTCTCTTGTATCGG |
| qATG13a-F | TCTCGTGGTCGTACTCGTGAG |
| qATG13a-R | TATCGTTTGAAATCGGATGGAC |
| qATG13b-F | GCAGGAGGTCAAGCAGTAATAAG |
| qATG13b-R | AACCGATAAGCAAAGTCTACCA |
| qATG8a-F | CGAATCGCAATGGCTAAGAGTT |
| qATG8a-R | CATCAATGCAGCAGTTGGAGG |
| qATG8e-F | ACCTTGTGCCATCAGACCTAAC |
| qATG8e-R | AGCCATCTTCGTCTTTCTTATCC |
| gnoCOR47-F | catgattacgaattcgagctcTTTATGAATCTATAGTATATTTGTTCATAAACG |
| gnoCOR47-R | caggtcgactctagaggatccATCATCAGACTCTTTTTCTTTCTTCACT |
| proATG8a-F | agtgccaagctctagttaattaaCTTTTCAATGTTATATAGCAACTAGACATG |
| proATG8a-R | cccggggatcgatcctctagaAATTAATAAACTCGATCGTCTGCTAGA |
| proATG13a-F | agtgccaagctctagttaattaaAATATTTTCTCCTTACTTGAAACTTAAACA |
| proATG13a-R | cccggggatcgatcctctagaCTTCTTCACACAAATCCCTAAATCG |
