# Supplementary figures and images for: Autophagy deficiency confers freezing tolerance in Arabidopsis thaliana
Source: BMC Plant Biol. 2025 Jul 30;25:994. doi: 10.1186/s12870-025-07066-9 (PMC12312434; doi:10.1186/s12870-025-07066-9)

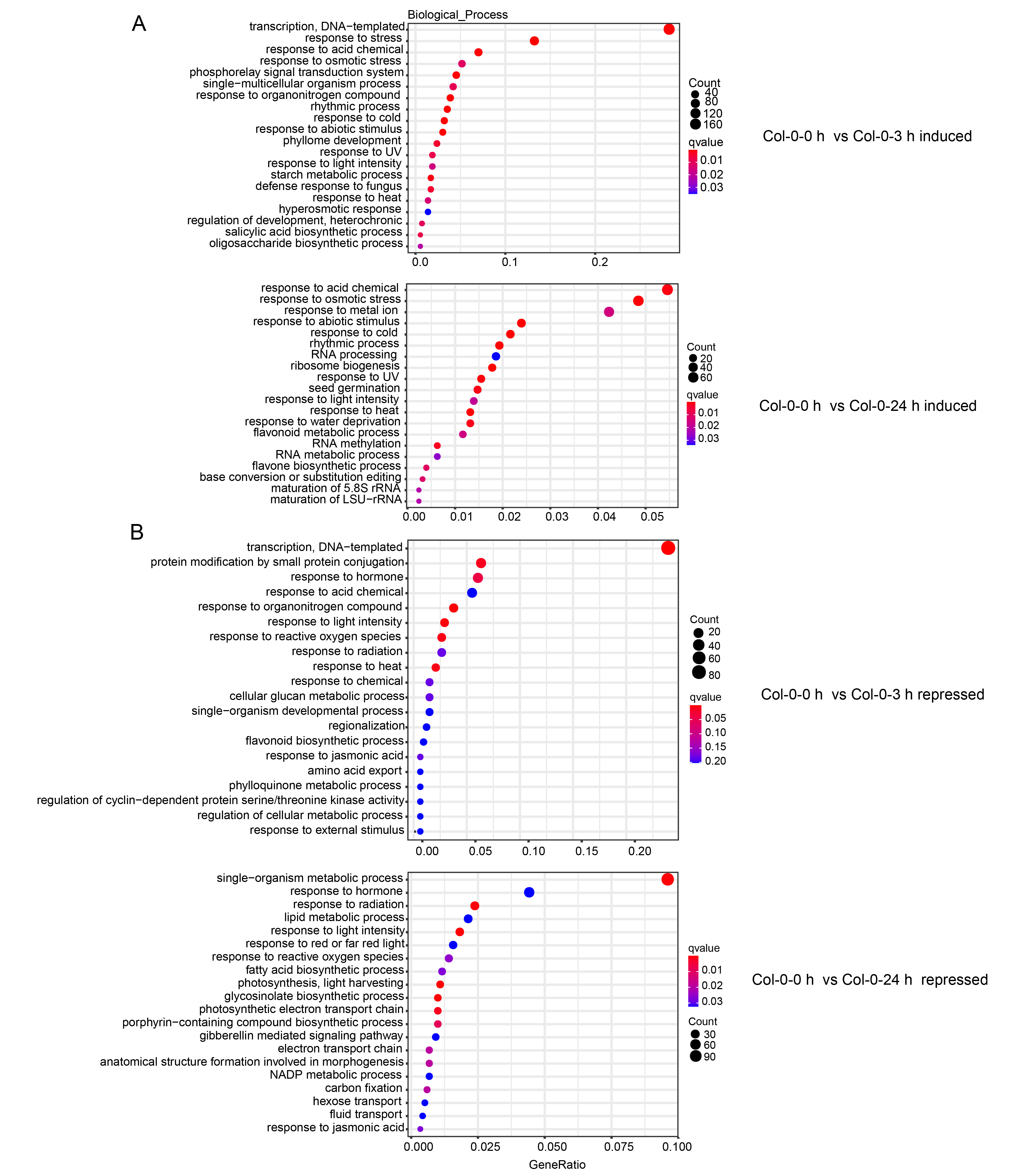

Supplement: Supplementary file 4 — Supplementary Material 4 [file 12870_2025_7066_MOESM4_ESM.jpg]

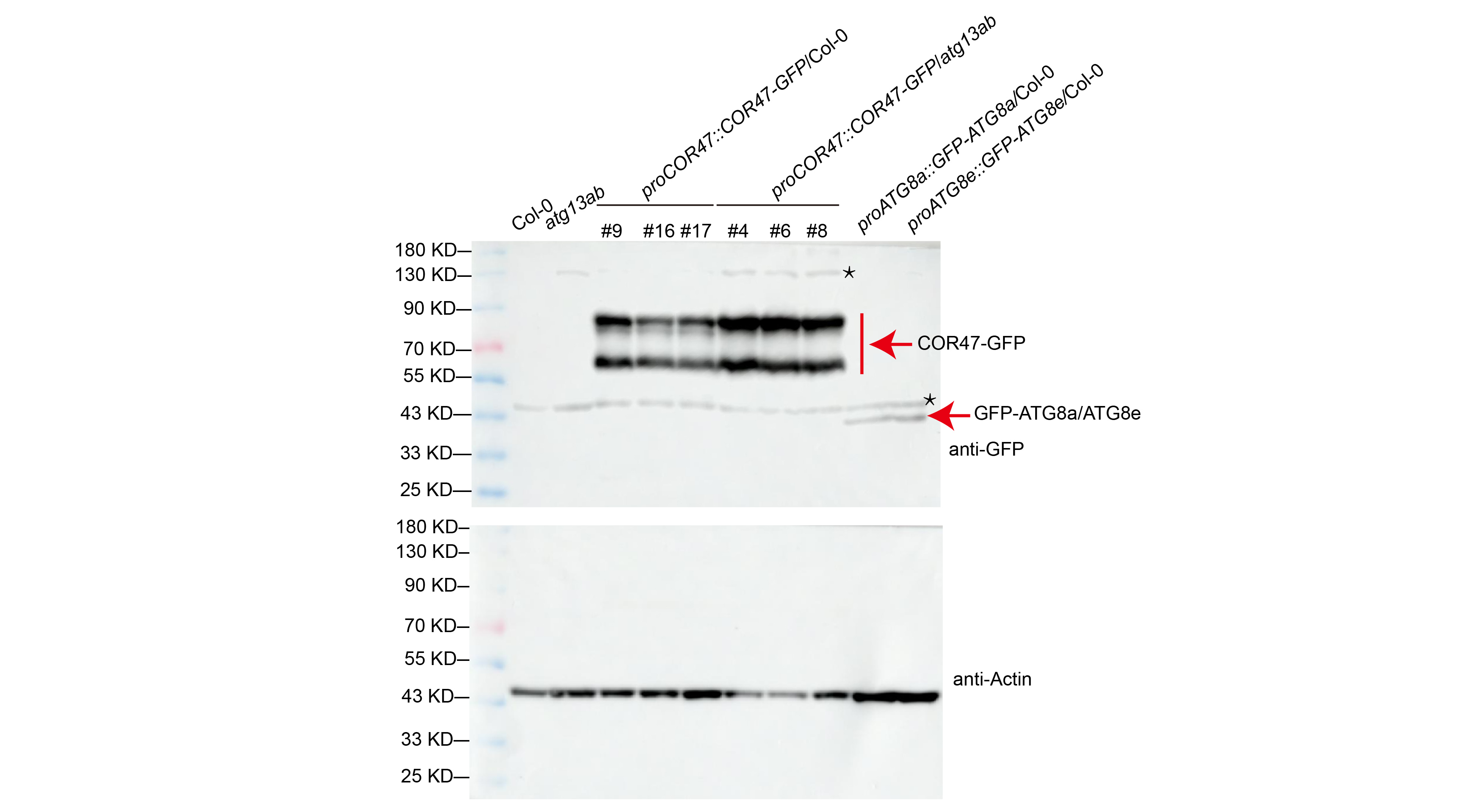

Supplement: Supplementary file 5 — Supplementary Material 5 [file 12870_2025_7066_MOESM5_ESM.jpg]

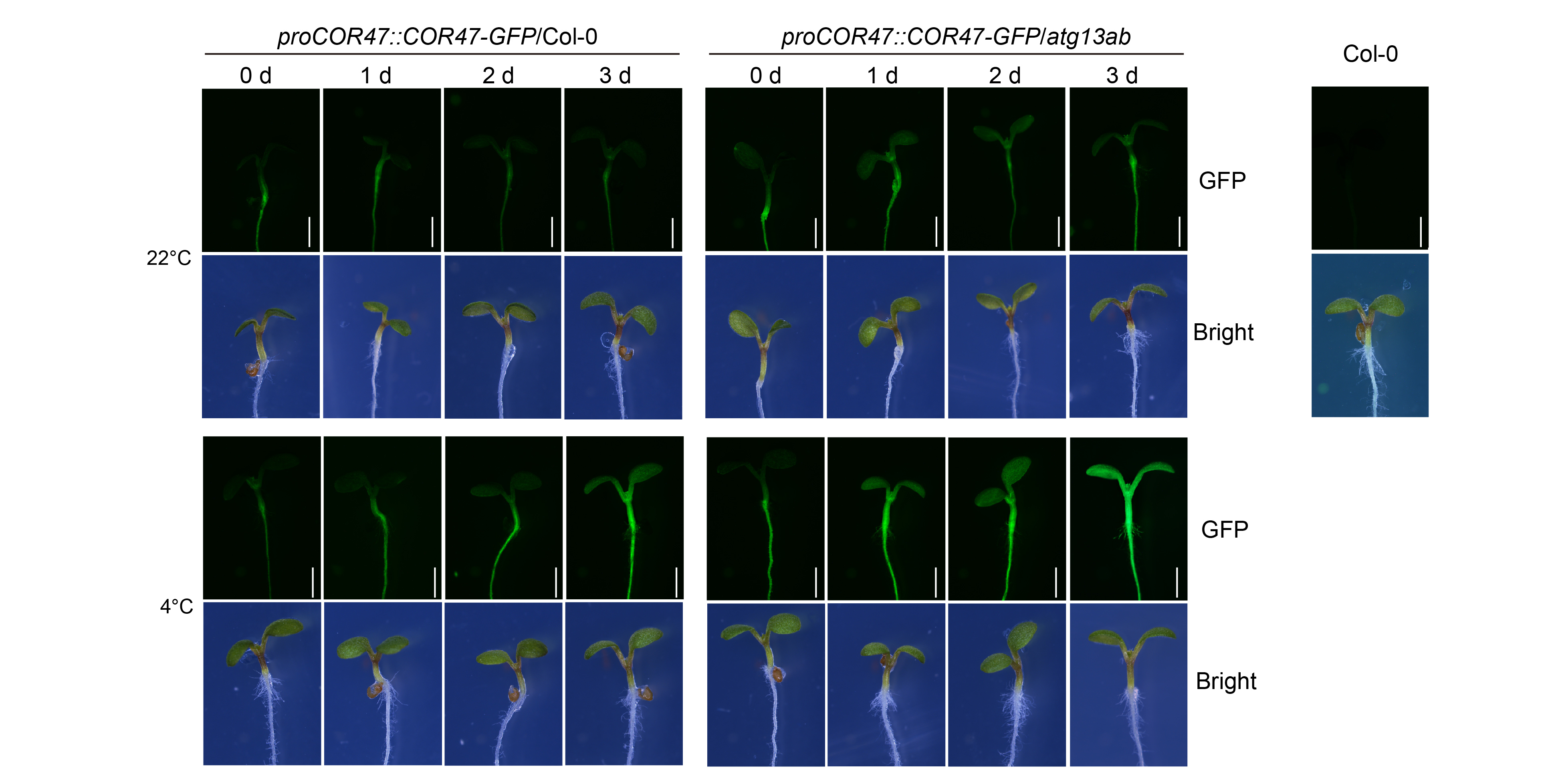

Supplement: Supplementary file 6 — Supplementary Material 6 [file 12870_2025_7066_MOESM6_ESM.jpg]

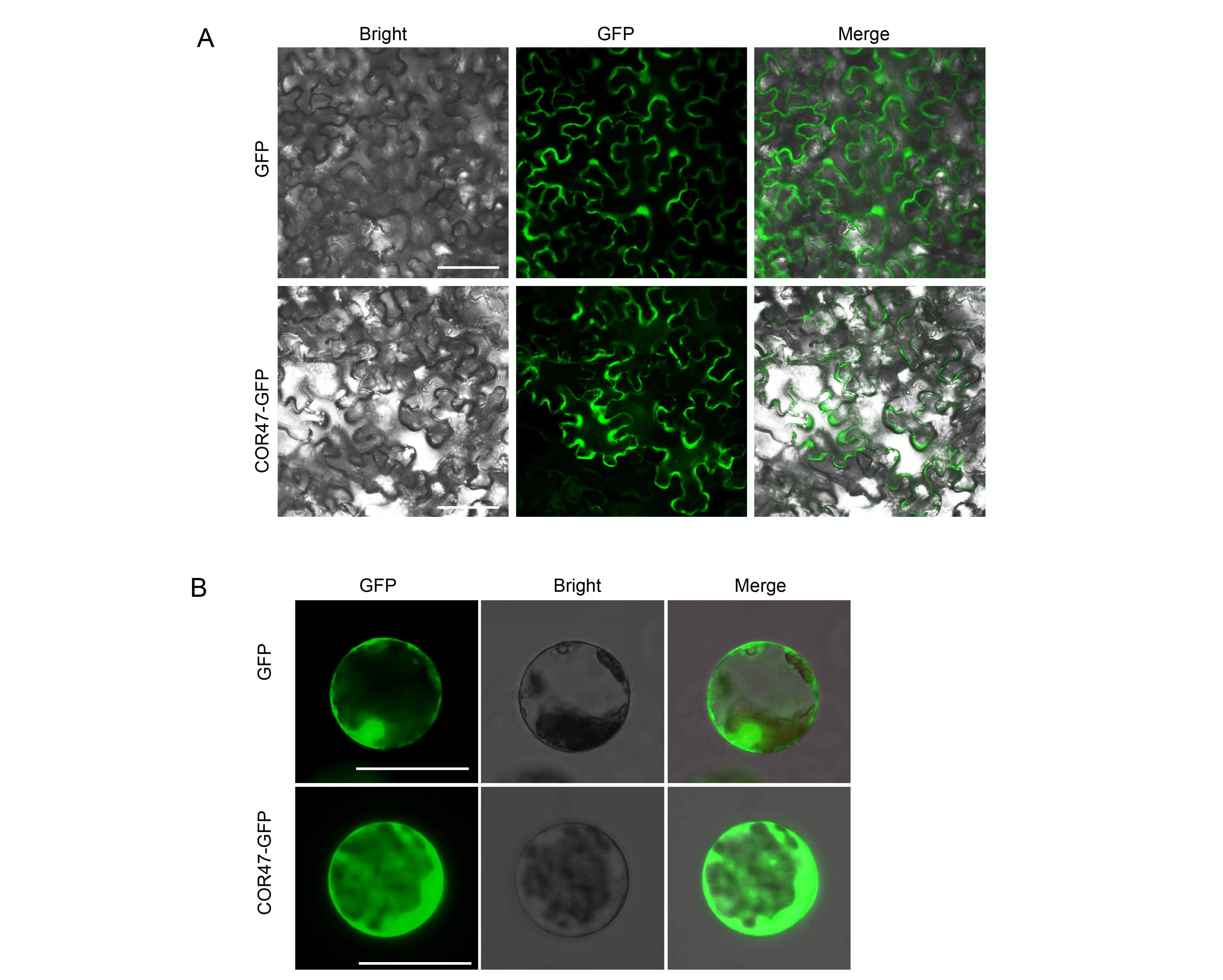

Supplement: Supplementary file 7 — Supplementary Material 7 [file 12870_2025_7066_MOESM7_ESM.jpg]

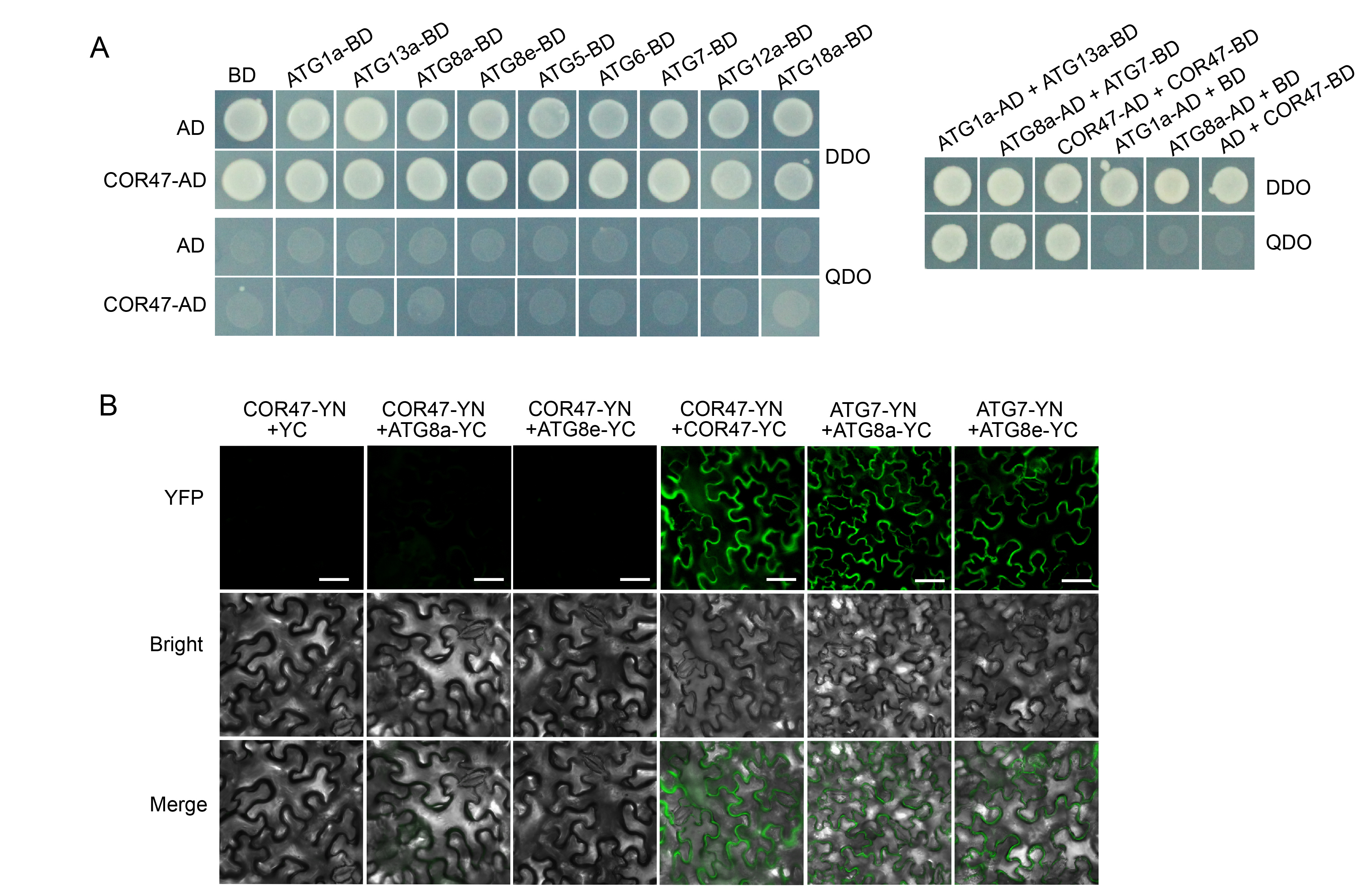

Supplement: Supplementary file 8 — Supplementary Material 8 [file 12870_2025_7066_MOESM8_ESM.jpg]
